# Synthetic biology tools for engineering Goodwin oscillation in *Trypanosoma brucei brucei*

**DOI:** 10.1016/j.heliyon.2022.e08891

**Published:** 2022-02-03

**Authors:** Yanika Borg, Sam Alsford, Vasos Pavlika, Alexei Zaikin, Darren N. Nesbeth

**Affiliations:** a The Advanced Centre for Biochemical Engineering, Department of Biochemical Engineering, Bernard Katz Building, Gordon Street, University College London, London, WC1E 6BT, UK; b Department of Mathematics and Institute for Women's Health, University College London, Gower Street, London, WC1E 6BT, UK; c Faculty of Infectious and Tropical Diseases & Department of Infection Biology, The London School of Hygiene & Tropical Medicine, Keppel Street, London, WC1E 7HT, UK; d World-Class Research Center “Digital Biodesign and Personalized Healthcare”, Sechenov First Moscow State Medical University, Moscow, Russia; e Lobachevsky State University of Nizhny Novgorod, Nizhny Novgorod, Russia

**Keywords:** Synthetic biology, *Trypanosoma brucei*, Live cell imaging, Synthetic gene networks, Oscillation

## Abstract

Kinetoplastid protozoa possess properties that are highly divergent from the mammalian, yeast and bacterial cells more commonly used in synthetic biology and represent a tantalisingly untapped source of bioengineering potential. *Trypanosoma brucei brucei* (*T. b. brucei*), an established model organism for studying the Kinetoplastida, is non-pathogenic to humans and provides an interesting test case for establishing synthetic biology in this phylogenetic class. To demonstrate further the tractability of Kinetoplastida to synthetic biology, we sought to construct and demonstrate a Goodwin oscillator, the simplest oscillatory gene network, in *T. b. brucei* for the first time. We report one completed iteration of the archetypal synthetic biology Design–Build–Test–Learn (DBTL) cycle; firstly, using *Ab initio* mathematical modelling of the behaviour a theoretical, oscillatory, trypanosomal synthetic gene network (SGN) to inform the design of a plasmid encoding that network. Once assembled, the plasmid was then used to generate a stable transfectant *T. b. brucei* cell line. To test the performance of the oscillatory SGN, a novel experimental setup was established to capture images of the fluorescent signal from motion-restricted live cells. Data captured were consistent with oscillatory behaviour of the SGN, with cellular fluorescence observed to oscillate with a period of 50 min, with varying amplitude and linear growth trend. This first DBTL cycle establishes a foundation for future cycles in which the SGN design and experimental monitoring setup can be further refined.

## 1 Introduction

**Figure 1:**
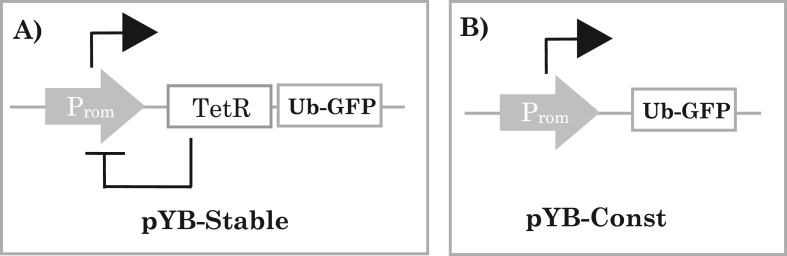
Goodwin oscillator concept. A) Overview of the minimal Goodwin oscillator, encoded within the plasmid, pYB-Stable. A constitutively active promoter (grey arrow box labelled ‘P_rom_’) will initially drive transcription of a repressor protein, in this case TetR. Once produced, the TetR protein will homodimerise and bind to a repressor binding site positioned to prevent further transcription driven by the original promoter - indicated by the hammer head connector from the TetR open reading frame (ORF) to P_rom_. Once it is no longer being replenished, TetR protein will eventually unbind the repressor site as it destabilises due to its inherent level of stability and the effect of surrounding cellular processes. When the repressor site is unbound, the constitutive promoter is free to re-initiate expression of TetR once more. Positioning an ORF for a short half-life GFP variant, Ub-eGFP, downstream of the TetR ORF, could enable the activity of the P_rom_ to be monitored experimentally as fluorescence should rise and fall in response to P_rom_ to activity. **B)** Overview of the control construct, pYB-Const, encoding a control gene in which expression of Ub-eGFP will be constitutive as no repressor is present.

Goodwin (1963) proposed the simplest genetic oscillator (Figure 1) as a single gene that auto-represses its own expression. Mathematical models of the oscillatory behaviour of the Goodwin oscillator (Griffith 1968; Tyson and Othmer 1978) have been applied extensively to predict the behaviour of synthetic (Novak and Tyson 2008; Purcell et al., 2010) and natural bio-oscillatory systems such as circadian rhythms (Gonze et al., 2005; Ruoff et al., 2001). Theoretical studies of synthetic gene oscillators have provided algorithms for their design (Chen and Chen 2010; Chang et al., 2013), which can model the impact of oscillatory output on cell division (Gonze, 2013), growth rate (Osella and Lagomarsino 2013), nutrient availability, intra- and extra-cellular conditions (O'Brien et al., 2012) and quorum sensing (Chen and Hsu 2012; Lang et al., 2011; Garcia-Ojalvo et al., 2004).

A growing body of research has been reported on the design and characterisation of synthetic gene oscillators. A diverse range of oscillatory dynamics have been observed, with oscillatory period cycles spanning 13 min (Stricker et al., 2008) to 26 h (Tigges et al., 2010), non-sinusoidal relaxation oscillations with steep amplitude rises and gradual decreases (Tigges et al., 2010; Atkinson et al., 2003), as well as classical sinusoidal oscillation patterns (Danino et al., 2010; Stricker et al., 2008). Biological noise, such as the non-synchronicity and range of transcription and translation rates between genetically identical cells (Tsimring 2014), has been suggested as the cause of stochasticity, amplitude variability (Elowitz and Leibler 2000) and amplitude dampening (Fung et al., 2005; Atkinson et al., 2003).

## 2 Novel chassis for synthetic biology

Most synthetic biology research to date utilises the three archetypal workhorse cells of biotechnology: *Escherichia coli* (*E. coli*), *Saccharomyces cerevisiae* and immortalised mammalian cells (Saito and Yokobayashi 2019), in anticipation of rapid industrial application. While alternative bacterial (Hoff et al., 2020) and budding yeast host cells (Henríquez et al., 2020) continue to be exploited by synthetic biologists, longer term goals include the eventual *de novo* design of genomes to control bacterial (Robertson et al., 2021), yeast (Ong et al., 2021) and even mammalian (Greene et al., 2019) cellular ‘chassis’. Against this background the protozoa present an interesting opportunity for synthetic biologists. The protozoa are highly evolutionarily divergent from the archetypal biotechnology workhorse organisms, often encoding genomic organisational properties, morphological phenotypes, metabolic networks and signal transduction pathways that represent potential new bioengineering capabilities.

The Kinetoplastida are a protozoan class within the phylum Euglenozoa, supergroup Excavata, characterised by possession of a flagellum and a kinetoplast, a dense, DNA-containing structure present within a lone, extended mitochondrion. The Kinetoplastida includes genera of parasites found in mammals, insects, birds and plants (Jackson 2014). The Kinetoplastid *Leishmania tarentolae* is already an established niche recombinant protein production platform (Klatt et al., 2019). Mol et al. (2018) have paved the way for applying synthetic biology approaches to *Leishmania major* by building synthetic networks in the organism to investigate the aetiology of the pathology it can cause in humans.

*Trypanosoma brucei brucei* (*T. b. brucei*) has been used extensively as a model Kinetoplastid organism and is the most widely studied organism in the Excavata supergroup. An extensive body of techniques and molecular toolkits has been established for laboratory-based cultivation and genetic manipulation of *T. b. brucei*, including systems for rapid plasmid assembly, targeted transgene integration, RNA interference and CRISPR/Cas9 applications (Yagoubat et al., 2020).

*T. b. brucei* cells adopt four major morphological forms associated with different stage of their life cycle. Procyclic form cells are relatively elongated and proliferate in the midgut of insects. Upon populating insect salivary glands, for transfer to mammalian hosts, cell division stops, and cells adopt a shorter, metacyclic form. In the bloodstream of mammalian hosts, *T. b. brucei* cells proliferate as long slender forms and upon receipt of a quorum sensing signal differentiate into non-dividing, short, stumpy forms pre-adapted for transmission to the Tsetse fly (Cayla et al., 2019). Both the long slender bloodstream and insect stage procyclic forms can be cultivated in vitro.

Within mammalian hosts, bloodstream form (BSF) *T. b. brucei* express a variant surface glycoprotein (VSG) that coats the cell surface (Smith et al., 2009). Periodically and stochastically, a sub-population arises that has switched VSG coat, enabling ongoing evasion of the immune response. The *T. b. brucei* genome contains more than 1000 VSG genes and pseudo genes, only one of which is expressed at a given time. The expressed VSG gene is located in one of approximately 15 sub-telomeric expression sites (Hertz-Fowler et al., 2008). Expression sites (ES) can be activated by in situ switching and new VSG genes generated through recombination with between ESs or with VSG genes and pseudo genes located in long arrays elsewhere in the genome. Each potential VSG protein is similar in structure but antigenically distinct (Hutchinson et al., 2007). Tight packing of VSG proteins on the cell surface also provides a barrier against innate immune responses (Schwede et al., 2015). In the insect host, procyclic *T. b. brucei* express procyclin proteins encoded by a small number of invariant genes. Procyclin coats the cell surface and is thought to protect against digestive enzymes in the insect gut (Acosta-Serrano et al., 2001).

## 3 Gene expression in trypanosomes

Transcription in trypanosomes is polycistronic (Breitling et al., 2002), with a single promoter site driving transcription of pre-mRNA molecules encoding multiple open reading frames (ORFs). The nascent transcript is processed to generate mature mRNAs following polyadenylation and trans-spicing, whereby the latter process adds a common 5’ cap-containing sequence to every mRNA (Ullu et al., 1993). In trypanosomes, as in other eukaryotes, RNA polymerase I transcribes non-protein encoding RNA, such as ribosomal RNA (rRNA), RNA polymerase II transcribes mRNA, and RNA polymerase III transcribes tRNAs, 5S rRNA and snRNAs (Palenchar and Bellofatto, 2006). Uniquely within studied eukaryotes, *T. b. brucei* RNA polymerase I also transcribes the mRNA for the surface coat proteins, procyclin and VSG (Ooi and Rudenko, 2017). RNA polymerase I-mediated VSG mRNA transcription occurs in an extra-nucleolar structure (Navarro and Gull 2001) known as the Expression Site Body (ESB). RNA polymerase-I mediated monoallelic VSG expression is dependent on the VSG exclusion (VEX) complex, which comprises VEX1, VEX2 and the histone chaperone, CAF1B (Glover et al., 2016; Faria et al., 2019). Remarkably, the RNA polymerase I mediated transcription machinery within the ESB transcribes the expressed VSG at approximately 50 times the rate of essential housekeeping β tubulin genes (Park et al., 2011).

## 4 Synthetic biology and trypanosomes

Synthetic biology is a relatively new field which can be defined as a multidisciplinary effort to make living biological material easier to engineer (Elgabry et al., 2020), enabling new products or functions that are not produced by natural biology. Studies in the field, such as this one, may advance the tractability of biology to engineering approaches as a foundation for future, as yet unidentified functions or products. As such, synthetic biology research often involves the implementation of genetic circuits in cells using standard molecular biology tools (Tigges et al., 2010), testing whether those circuits function as expected, and using mathematical models to gain insight, inform troubleshooting and design future iterations of a given circuit. A key requirement for engineering biology is the capture and accessible provision of metabolic data sets to enable construction of accurate mathematical models. For trypanosomes the development of the TrypanoCyc (www.metexplore.fr/trypanocyc/) database of metabolic pathways has been particularly valuable. The ‘silicon trypanosome’ project made progress toward an in-depth in silico mathematical model of a trypanosome (Bakker et al., 2010) and utilised a previous model of the metabolic pathways of bloodstream form trypanosomes (Bakker et al., 1997). In this work we aimed to establish for the first time the viability of *T. b. brucei* as a chassis system for synthetic biology by completing one iteration of the archetypal synthetic biology Design–Build–Test–Learn (DBTL) cycle to establish an oscillatory, trypanosomal SGN. This first DBTL cycle required development of mathematical models to predict gene expression and protein stability in *T. b. brucei*, design and construction of a gene network to embody the oscillations and establishment of an experimental setup to monitor fluorescence in live cells, all of which we report here.

## 5 Materials and Methods

### 5.1 Plasmid assembly

Standard molecular biology techniques were used for all DNA procedures, with all enzymes purchased from New England Biolabs, USA. Full details are also available in Borg (2015), in which plasmids pYB-Stable and pYB-Const are referred to as ‘pStable’ and ‘pConstitutive’ respectively. For assembly of the pYB-Const plasmid, firstly a DNA fragment was designed encoding an amino-terminal (N-terminal) ubiquitin-e^K^ degron domain, referred to here as UbeK, flanked upstream of the start codon by a *Hind III* site, with an in-frame *Xba I* site spanning the end of the protein-coding region. The UbeK degron sequence is available at Borg et al. (2021a) and was synthesised by Eurogentec (Southampton, UK) in a pUC57 backbone within the plasmid, pUbeK.

The trypanosome expression plasmid, pRP_eGFP_SIR2rp3, kindly provided by Prof. David Horn (sequence available on request), and pUbeK were used in a three-fragment ligation attempt (Figure 6A) which included mutagenic PCR of pRP_eGFP_SIR2rp3, with forward primer TCTagaGTGAGCAAGGGCGAGGAGCTGTTCACCGGGG, and reverse primer: GGATCCGCCTTCaAGACTTGTACAGCTCGTCC, to amplify a fragment partially encoding an enhanced green fluorescent protein (eGFP) ORF. The forward primer was designed to remove a start codon and introduce an in-frame *Xba I* site, indicated by lower case and underlined bases respectively, and the reverse primer to introduce a stop codon and replace an *Xba I* site with a *Bam HI* site, indicated by lower case and underlined bases respectively. In separate reactions, *Hind III* and *Bam HI* were used to digest pRP_eGFP_SIR2rp3 to provide a backbone without the eGFP-SIR2rp3 fusion protein ORF and to digest pUbeK to provide a fragment encoding a the UbeK ORF with an in-frame *Xba I* overhang end. This ligation yielded the plasmid, pUbSir, as an unintended product encoding an ORF for an UbeK-SIR2rp3 fusion protein.

A two-fragment ligation by Gibson assembly (Gibson et al., 2009) was then performed using a round of PCR with pRP_eGFP_SIR2rp3 as template with forward primer CTAGACAAGTTTCTAGAGTGAGCAAGGGCGAGGAG and reverse primer GCCAACTAAATGGGCAGGATCCGCCTtcaAGACTTG, to generate a fragment encoding a partial eGFP ORF with no start codon and only a stop codon, indicated by lower case bases in reverse primer, and regions identical to the intended insertion site within the destination plasmid, underlined. PCR was also performed with pUbSir as template with primers GGATCCTGCCCATTTAGTTGGC and TCTAGAAACTTGTCTAGCC to amplify the plasmid without the SIR2rp3 region of the UbeK-SIR2rp3 ORF. These two PCR products were joined to generate the pYB-Const plasmid (Figure 6B).

A final round of PCR was performed with the plasmid pHD1313, kindly provided by Prof. Christine Clayton (Center for Molecular Biology, Heidelberg, Germany), as template using primers CAATGATAGAGTGGTACCGTCTTGGTGTGTCGACCTTG and GCGCGTGCAGGGTACCTTGTACATATTGTCGTTAGAACGCC to generate a fragment encoding a TetR ORF flanked upstream by an EP1 5′ untranslated region (UTR), downstream by the ΔALD 3’ UTR and with regions identical to the intended insertion site within the destination plasmid, underlined. pYB-Const was linearised by digestion of a lone *Kpn I* site present directly downstream of the TetO sequence. The PCR fragment and *Kpn I*-linearised pYB-Const were ligated to generate the pYB-Stable plasmid (Figure 7).

### 5.2 *T. b. brucei* cultivation

The procyclic form (PCF) Lister 427 monomorphic line (Cross 1975) was used throughout. Cultivation of PCF cells was performed using semi-defined SDM-79 media (Schonenberger 1979). 4.5 *L media* was prepared using SDM-79 powder (Life Technologies) supplemented with 10 g sodium bicarbonate (Sigma Aldrich, Munich, Germany) and 50 mL of ‘Pen-Strep’ penicillin and streptomycin solution (Life Technologies). The solution was filter-sterilised and stored in 450 mL volumes at 4 °C. Prior to use, 50 mL 56 °C heat-inactivated FBS and 1.5 mL 2.5 mg/mL hemin (Sigma Aldrich) was added to each 450 mL aliquot. The media was then stored at 4 °C and pre-warmed to 28 °*C prior* to use. Cells were maintained at a minimum density of 1 × 10^6^-2×10^7^ cells/mL, in non-ventilated flasks (Fisher Scientific, MA. USA), in an LMS Series Two Cooled Incubator (LMS Ltd., Kent, UK) at 28 °C.

Cells were cryopreserved by addition of 100 μL of 100% v/v glycerol to 900 μL of culture at a minimum of 1×10^7^ cells/mL. This was then stored at -80 °C for 24 h in a Nalgene Mr Frosty device and then transferred to liquid nitrogen. For revival 1 mL vials were thawed on ice and the contents were added to 10 mL pre-warmed media, centrifuged for 10 min at 2000 revolutions per minute (rpm) using an Eppendorf Centrifuge 5804 (Eppendorf, Stevenage, UK). This centrifuge was used for all procedures involving *T. b. brucei* cells in this report. Supernatant was then removed and the cell pellet was resuspended in 2 mL media prior to incubation at 28 °C. Following the 24 h incubation period, selective antibiotic, if appropriate, was added.

To maintain stable transfectants the media was supplemented with hygromycin (Sigma Aldrich) at 2.5 μg/mL, prepared from 5 mg/mL in distilled water (dH_2_O) stock, which had been filter-sterilised and stored at -20 °C. For gene induction a 1 mg/mL tetracycline stock solution was prepared by dissolving 10 mg of tetracycline in 10 mL dH_2_O, followed by filter-sterilisation and storage at -20 °C. Tetracycline supplementation was then performed at the concentrations indicated in the Results section. Unless otherwise specified, cultures were incubated in the presence of indicated tetracycline concentrations for 24 h prior to harvesting or analysis.

### 5.3 Stable *T. b. brucei* transfection

Prior to transfection, 10 μg of plasmid insert DNA was linearised with *Not I* and purified using standard molecular biology techniques. Conditioned medium was freshly prepared prior to each transfection attempt. For production of conditioned media, 200 mL PCF cells were grown to a density of 1×10^7^ cells/mL. This volume was then divided into 50 mL aliquots and centrifuged at 2000 rpm for 10 min. After this, the supernatant was retained, transferred to a new tube and the centrifugation was repeated. Again, the supernatant was retained, after which it was passed through an 0.2 μm filter (Acrodisc® Syringe Filter) with 0.2 μm Supor® Membrane (Pall Life Science, Portsmouth, UK) and stored at 4 °C.

1×10^7^ PCF cells were centrifuged at 2000 rpm for 10 min (Alsford and Horn 2012). Supernatant was removed, the cells were resuspended in 0.5 mL phosphate buffered saline (PBS) solution (Sigma-Aldrich) and the cell suspension was centrifuged for 1 min at 10,000 rpm. The supernatant was again removed, and cell pellet was resuspended in 100 μL Cytomix plus 10 μL linearised DNA. Cytomix consisted of 120 mM KCl, 25 mM 4-(2-hydroxyethyl)-1-piperazineethanesulfonic acid (HEPES) at pH 7.6, 0.5% w/v glucose, 100 μg/mL bovine serum albumin, 150 μM CaCl_2_, 2 mM ethylene glycol-bis(β-aminoethyl ether)-N,N,N′,N'-tetraacetic acid (EGTA) at pH7.6, 1 mM hypoxanthine, 5 mM MgCl_2_.6H_2_O and 10mM K_2_HPO_4_/KH_2_PO_4_ at pH7.6. All of the cytomix solution of cells and linearised DNA was then transferred to a 0.2 cm electrocuvette for electroporation in an Amaxa™ Nucleofector™ II device (Lonza Group Ltd., Basel, Switzerland) using nucleofection programme X-001. Following transfection, 1 mL of pre-warmed media was added to the cuvette and all cuvette contents transferred to 60 mL pre-warmed growth media in a cell culture flask. The flask was then incubated for 6 h in standard conditions to allow time for expression of the hygromycin resistance gene within the plasmid. 140 mL, pre-warmed, conditioned (Archer, 2009) media, supplemented with 10% v/v 56 °C heat-inactivated FBS, was then added and the cell suspension was mixed by gentle swirling. *A* 1 mL *aliquot of this cell solution was used for cell counting which informed serial dilution to achieve a 2.5 cells/mL solution.* 200 μL *of this cell solution was then transferred to each well of a 96-well plate, to give a predicted cell count of 0.5 cell/well.* Five days post plating, wells populated with growing cells were identified and their contents transferred to 10 mL pre-warmed, hygromycin-supplemented media as uniquely labelled clones. Cultivation under hygromycin selection then continued until sufficient cells were available for glycerol-based cryopreservation. Growth characteristics of the *T. b. brucei* PCF parental strain and the clonally derived transfectant strain TbGOS02 were determined by inoculating media at 2 × 10^6^ cells/mL, in duplicate, followed by incubation for three days with twice daily cell counts using a Coulter counter. Doubling time, calculated by linear regression of growth data, was 10.1 h for the parental strain and 4.9 +/- 4 h for strain TbGOS02, which decreased to 7.6 +/- 2.2 when cells were grown in the presence of 10 μg/mL tetracycline.

### 5.4 Protein electrophoresis and Western blotting

10 mL aliquots of *T. b. brucei* PCF cells at a minimum concentration of 5×10^5^ cells/mL were centrifuged at 2000 rpm for 10 min, the supernatant was removed and the cell pellet resuspended in 10 mL PBS. This centrifugation and resuspension was repeated once followed by final resuspension in 500 μL PBS. A 2-min centrifugation at 5000 rpm was then applied, supernatant removed and cell pellet resuspended in 100 μL of a sample buffer: 62 mM Tris, 10% v/v glycerol, 2.3% w/v sodium dodecyl sulphate (SDS), 5% w/v β-mercaptoethanol, 0.002% w/v bromophenol blue (Sigma Aldrich), vortexed for 2 s then stored at -20 °C. 5 × 10^6^–2x10^7^ cells per lane were electrophoresed through 15% SDS-PAGE minigels alongside PageRuler Prestained Protein Ladder (Fisher Scientific) or the Precision Plus Protein™ WesternC™ Standard (Bio-Rad) ladder using an Electrophoresis Power Supply (Fisher Scientific) power pack. The gel was placed into fixing solution; 25% v/v isopropanol (VWR International Ltd., Leicestershire, U.K), 10% v/v acetic acid (Fisher Scientific) in distilled water, and rotated at 20 rpm for 15 min using the Stuart Mini See-Saw Rocker SSM4 (Bibby Scientific Ltd., Staffordshire, UK) then placed for 1 h in staining solution; 10% v/v acetic acid, 60 mg/L Coomassie brilliant blue G-250 (Sigma Aldrich) in distilled water, under 20 rpm rotation. Finally, the gel was placed in a de-staining solution, 10% v/v acetic acid in distilled water, and rotated at 20 rpm for 30 min. A GeneGenius Bio-Imaging System (Syngene, Cambridge, UK) apparatus was used with the image acquisition software GeneSnap (v. 6.08, Syngene, Cambridge, UK) to capture gel images.

For Western blotting, SDS-PAGE gels were first placed in transfer buffer; 5.83 g/L Tris, 2.93 g/L glycine, 20% v/v methanol, 375 mg/L SDS in 1 L dH_2_0, for 15 min under rotation at 20 rpm. Two sheets of Extra Thick Blot Paper™ (Bio Rad) were soaked in transfer buffer for at least 10 min, with no agitation. A 0.45 μm nitrocellulose membrane (Hybond, Amersham Biosciences) was placed in transfer buffer for 30 s, transferred to water that had been purified by reverse osmosis (rH_2_O) for a further 1 min and then placed again into transfer buffer for 10 min, this time with 20 rpm agitation. Membrane and filter paper were then placed into a Trans-Blot® Turbo™ Blotting System (Bio-Rad) apparatus as part of a sandwich, with filter paper outermost and the gel on top of the filter paper, above the base anode cassette. The Bio Rad pre-defined Standard SD program was used, where a voltage of 25 V and a current of 1 Amp were passed through the transfer sandwich for 30 min.

Following transfer, the membrane was placed in blocking solution; 5% v/v milk powder (Marvel, Premier International Food UK Ltd., Lincolnshire, UK) 0.05% v/v TWEEN®-20 (Sigma Aldrich) in PBS for eGFP detection and in Tris-Buffered Saline solution (TBS), 50 mM Tris at pH 8.0, 150 mM NaCl, for TetR detection.

After blocking, the membrane was placed in fresh blocking solution with a 1:5000 dilution of the anti-GFP Rabbit IgG Polyclonal Antibody Fraction product (Life Technologies) and incubated for 1 h with 20 rpm agitation. Following primary antibody incubation, the membrane was placed in fresh blocking solution under rotation at 20 rpm for 10 min. The washing procedure of blocking solution removed, replacement with fresh and incubation under rotation at 20 rpm for 10 min, was performed twice. Membrane was then placed in TBS-T (TBS with 0.05% v/v Tween 20) for a 5-minute incubation under 20 rpm rotation. The membrane was then placed in TBS-T plus 0.5% w/v milk powder with a 1:12000 dilution of Goat Anti-Rabbit IgG (H + L)-horseradish peroxidase (HRP) Conjugate (Bio-Rad) as secondary antibody and incubated at 20 rpm for a further 1 h. Membrane was removed from the secondary stain solution and placed in fresh TBS-T for 5 min at 20 rpm. This rinse process was performed twice.

Chemiluminescent imaging was achieved with the Amersham™ ECL™ Western Blotting Analysis system kit (GE Healthcare Life Sciences, Buckinghamshire, UK). Amersham™ Hyperfilm ECL autoradiography film (GE Healthcare Life Sciences) was pressed onto the membrane for an exposure of 15–60 s and the Compact X4 X-Ray film processor (Xograph Healthcare, Gloucestershire, UK) used to visualise images.

### 5.5 FILT setup for live cell imaging

A novel setup for measuring fluorescence in live trypanosome (FILT) cells was established as part of this study. All live-cell imaging experiments with *T. b. brucei* were performed in compliance with Specified Animal Pathogens Order 2008 (SAPO) License number PATH/151/2010/1, issued by the UK Department for the Environment, Food and Rural Affairs (DEFRA). Thermoreversible Cygel™ (BioStatus Ltd., Leicestershire, U.K) was stored at 4 °C and diluted in PBS prior to use. A 0.5 mL aliquot of cells that had been cultivated to a minimum concentration of 1×10^6^ cells/mL was centrifuged for 1 min at 5000 rpm, supernatant removed and cells resuspended in growth media to a concentration of 5×10^7^ cells/mL in 50 μL. A 5 μL aliquot of this cell suspension was added to 5 μL fresh growth media supplemented with Cygel™ to 80% v/v, to give a cell concentration of 2.5×10^6^ cells/μL in 40% w/v Cygel™. 4 μL of this solution was transferred by pipette onto the surface of a 5 mm 12-well slide, on top of which a cover slip was placed and sealed with nail varnish in an attempt to thwart any unanticipated routes by which cells might be transferred to the environment.

An AxioPlan2 microscope, set up with the QImaging® Retiga-2000R Fast 1394 digital camera, was used to acquire consecutive fluorescent and brightfield images over time. For brightfield images the following settings were used: 2 ms exposure, Monochrome colour format, Zeiss Fluo Turret Filter 1, Zeiss TL Voltage of 5.4 V, gain of 1.8x and offset of 46 levels. For fluorescent images the following settings were used: 2.0 s exposure, Screen colour format, Zeiss Fluo Turret Screen, Zeiss TL Voltage of 0.0 V gain of 0.0x and offset of 0 levels. Prior to imaging, the surface of the cover slide over each sample was covered with a drop of immersion oil. In order to acquire both brightfield and fluorescent images of the cells, the Volocity® 3D Image Analysis Software, v. 6.3.0 (Quorum Technologies, Puslich, Canada) was used automate image capturing. Images were captured every 2 min over 2 h.

Images were analysed using the Volocity 3D Image Analysis. An image of a blank well, in which no cell solution was present under the coverslip and mineral oil drop, was used as the dark reference image for background correction to distinguish fluorescent cells and background noise. Following image acquisition and background correction, fluorescence intensity of individual cells was quantified using Volocity Quantitation software set to quantify only fluorescent spots of size 20-10,000 μm^2^. Volocity Quantitation analysis was confirmed visually by tracking a cell of interest manually over each image acquired to ensure that the software had measured the correct object in each case.

### 5.6 *Ab initio* simulation of the Goodwin gene oscillator

The Gillespie (1977) Direct Method stochastic simulation algorithm (Gillespie SSA) was used to generate stochastic time series simulations of the quantity of molecular components/species encoded by pYB-Stable over a span of 500–1000 min. The algorithm works by selecting an initial time-point t0 and simulating when a biochemical reaction next takes place and also which reaction takes place. Molecule numbers and timings were then updated and the process repeated. The decision of when the next reaction takes place and also which type of reaction takes places is based on the generation of two random numbers from a uniform distribution with parameters 0 and 1. This generates a trajectory of the molecular components. The algorithm scheme runs as follows:

Step 1) Given thermal and spatial homogeneity:

Set $t_{0}$ and $t_{final}$*.* Set $t=t_{0}$

Store initial molecule numbers for the *N* species, $X_{i}\left(t_{0}\right)\text{​ ​for }i\in 1,..N$

Store reaction rate values of the M reactions as $c_{j}\text{ for }j\in 1,..M$

Step 2) Calculate the propensity values $a_{j}\left(t\right)=h_{j}c_{j}\text{ ​ ​for ​ ​}j\in 1,..M$*,* where $h_{j}$ is the number of available molecules of the reactant species in reaction j

Step 3) Calculate $\alpha_{0}=\overset{\text{M}}{\underset{j=1}{\sum }}\alpha_{\text{j}}$

Step 4) Generate a random pair $\left(r_{1},r_{2}\right)$ from the standard uniform distribution U(0,1)

Step 5) Calculate $\tau =\frac{1}{\alpha_{0}}.ln\left(\frac{1}{r_{1}}\right)$

Step 6) Calculate k such that $\overset{k-1}{\underset{j=1}{\sum }}\alpha_{\text{j}}<r_{2}.\alpha_{0}\leq \overset{k}{\underset{j=1}{\sum }}\alpha_{\text{j}}$

Step 7) Update t=t+τ and $X_{i}\left(t\right)$ for ​ ​i∈1,...N to reflect the changes in the population from the execution of reaction k

Step 8) Go to Step 2 and repeat until $t>t_{final}$

To simulate the dynamics of the pYB-Stable-encoded SGN behaviour using the Gillespie SSA, the ABC-Sysbio 2.05 (Liepe et al., 2010) Python-based program was used. ABC-Sysbio 2.05 is written in the Systems Biology Markup Language (SBML) developed by Keating and Le Novere (2013). Ubuntu 12.04 (www.ubuntu.com) Linux platform was used to access a Dell PowerEdge C6100 server plus C410X GPU chassis for running the algorithm. This remote system uses the centOS 5.8 operating system with a Tesla M2050, M2090 and K20 GPU for which permission to access was kindly provided by Dr. Chris Barnes, UCL Research Department of Cell and Developmental Biology. The script: *abc-sysbio-sbml-sum* was used by running the command: *abc-sysbio-sbml-sum –files file.xml* where *file.xml* is a model of the Goodwin oscillator, in a terminal window. This command serves to parse and translate the SBML file of the model of the pYB-Stable-encoded SGN into an ABC Sysbio-compatible input ‘.xml’ file (input file template.xml). The *abc-sysbio-sbml-sum* script also inserts default settings in the file as a simulation of the species' quantity profiles. The file was then manually edited in order to specify the following algorithm and simulation conditions:

1. **<particles>** This represents the number of time series simulations to carry out.
2. **<data> <times>** The time points at which to print molecule numbers of the species which have been simulated.
3. **<models> <model1> <type>** The type of simulations to carry out. This was consistently set to Gillespie.
4. **<models> <model1> <parameters>** The distribution of the range of values which can be used for each parameter during simulations. The parameter values are randomly picked from these distributions at the beginning of each simulation. No simulation is carried out using the same set of parameters.
5. **<models> <model1> <initial>** The initial number of molecules for each of the species within the model.

Gillespie stochastic simulations were performed by running a *run-abc-sysbio* script as per the following command below, for which functions are defined in Table 1.*run-abc-sysbio –infile input file template.xml -f -of = res –timing –cuda –simulate*.

**Table 1:** Command functions.

Script component	*Request*
*-f*	Indicate when each iteration has finished
*-of*	Specify name of the folder in which to place simulation results
*–timing*	Print timing information
*–cuda*	use CUDA platform implementation
*–simulate*	generate time series for the specified model

Functions within the command: *run-abc-sysbio –infile input file template.xml -f -of = res –timing –cuda –simulate* used to perform Gillespie stochastic simulations of the putative Goodwin oscillator encoded by the plasmid pYB-Stable.

This script generates two text files: *particles.txt* which contains information about the parameter values which were selected from the user defined range of values and used in each simulation and *trajectories.txt* which lists the quantities of the different species at each of the specified time points in **<data> <times>**.

To carry out time series simulations, the SGN was represented as a set of molecular species and biochemical reactions. This information was coded in Copasi 4.12.65 (Hoops et al., 2006), which was used for debugging the mathematical model and for translating the data into SBML.

### 5.7 Data imputation

A moving average filter was applied to impute during the qualitative analysis process. Given a set of N data points x_*i*_*, ...,* x_*n*_*,* the moving average of data point *k, y*_*k*_*,* using window size *n* is based on the value of data point *k*, the previous *n* raw data points *x*_*i*_ and the subsequent n raw data points x_i_, as in Eq. (1.1).

(1.1) $$y_{k}=\;\frac{1}{2k+1}\overset{k+n}{\underset{i=k-n}{\sum }}x_{i}$$

given that *k n > 0* and *k + n ≤ N*. Otherwise, each data point with the index out of bounds is ignored, and the denominator subtracted by 1. This gives a set of *N* filtered data points *y*_*i*_*, ..., y*_*n*_*,*. By weighting each data point equally, the dominant trend is preserved while eliminating noise signals. This technique was implemented in MATLAB using a custom algorithm (Borg 2015).

### 5.8 Cubic spline data interpolation

A spline was used to fit a smooth curve to a set of data points. Given a dataset y with values defined at independent points t1, ..., tN, a cubic spline served to generate a stable piece-wise function, f:[a,b]!R where [a,b] = [a = t1 <t2 <...<tN 1 < tN = b], consisting of cubic polynomials between each consecutive pair of data points. The polynomials were continuous and continuously differentiable up to the second derivative, even at the interior boundary points (de Boor 1978). A cubic spline was fitted to the dataset of interest using the MATLAB function yy = spline (t, y) wherein yy is the output representing the coefficients of the spline's polynomials, t is the data array of independent variables taken to be time and y is the set of data points to which the spline must be fitted. Following this, v = ppval (pp,yy) was used to generate values of the piecewise polynomial function yy at time-points pp. The plot function was used to plot the fitted spline.

### 5.9 Linear growth trend fitting

The polyfit function in MATLAB was used to fit a given linear trend y = m.t + c to a dataset. The function works by running the command yy = polyf it (t, x, d) which fits a polynomial of degree d to the vector x using the vector t as an independent variable, generally taken to be time. In this case d = 1. The output yy generates a vector of coefficients representing m and c from the linear trend. To generate data points for the linear trend, y = polyval (pp,yy) was used wherein the coefficients of the polynomial in yy are used to generate a vector of values y at the vector of time-points pp.

### 5.10 Phase space reconstruction

A custom written program in MATLAB (Borg 2015) was used to reconstruct the phase space. Given a delay of k, the program used the plot (x, y) function to map the vector x against the vector y where y equals the vector x offset by k data points. N was the number of data points and x (i, 1) for i = 1, . . ., N was equal to the data points on which the reconstruction will be based, i.e. the variable output. Then the function plot (x (1 : (N k), 1), y (1 : (N k), 1)) was used where y (i, 1) = x (i + k, 1) for i = 1, . . ., N k.

## 6 Results and Discussion

### 6.1 *In silico* design of a trypanosomal Goodwin oscillator gene network

We decided to use the tetracycline repressor (TetR)/tetracycline operator (tetO) system to provide the negative feedback function within the trypanosomal Goodwin oscillator gene network design (Figure 1A). The TetR/tetO system has been implemented in many eukaryotic host cells (Gossen and Bujard, 1992; Kamionka et al., 2004), including multiple experimental studies in trypanosomes (Alibu et al., 2005; Peacock et al., 2005). The TetR protein ORF would be under transcriptional control of a strong, constitutive trypanosomal promoter designed to also features a tetO DNA sequence. Expressed TetR protein would dimerises and bind to tetO, repressing transcription from the promoter positioned upstream of the TetR ORF (Berens et al., 1992). When no longer replenished, TetR dimer would eventually be lost due to protein turnover, unblocking the promoter and rendering it available again for directing transcriptional activity. As a reporter of the status of the promoter and TetR expression, we designed the system to feature an ORF encoding a short half-life GFP variant, Ub-eGFP, also downstream of the strong promoter and TetR ORF, as part of a polycistronic expression cassette (Figure 1A).

### 6.2 Simulation of a trypanosomal Goodwin oscillator gene network design

**Figure 2:**
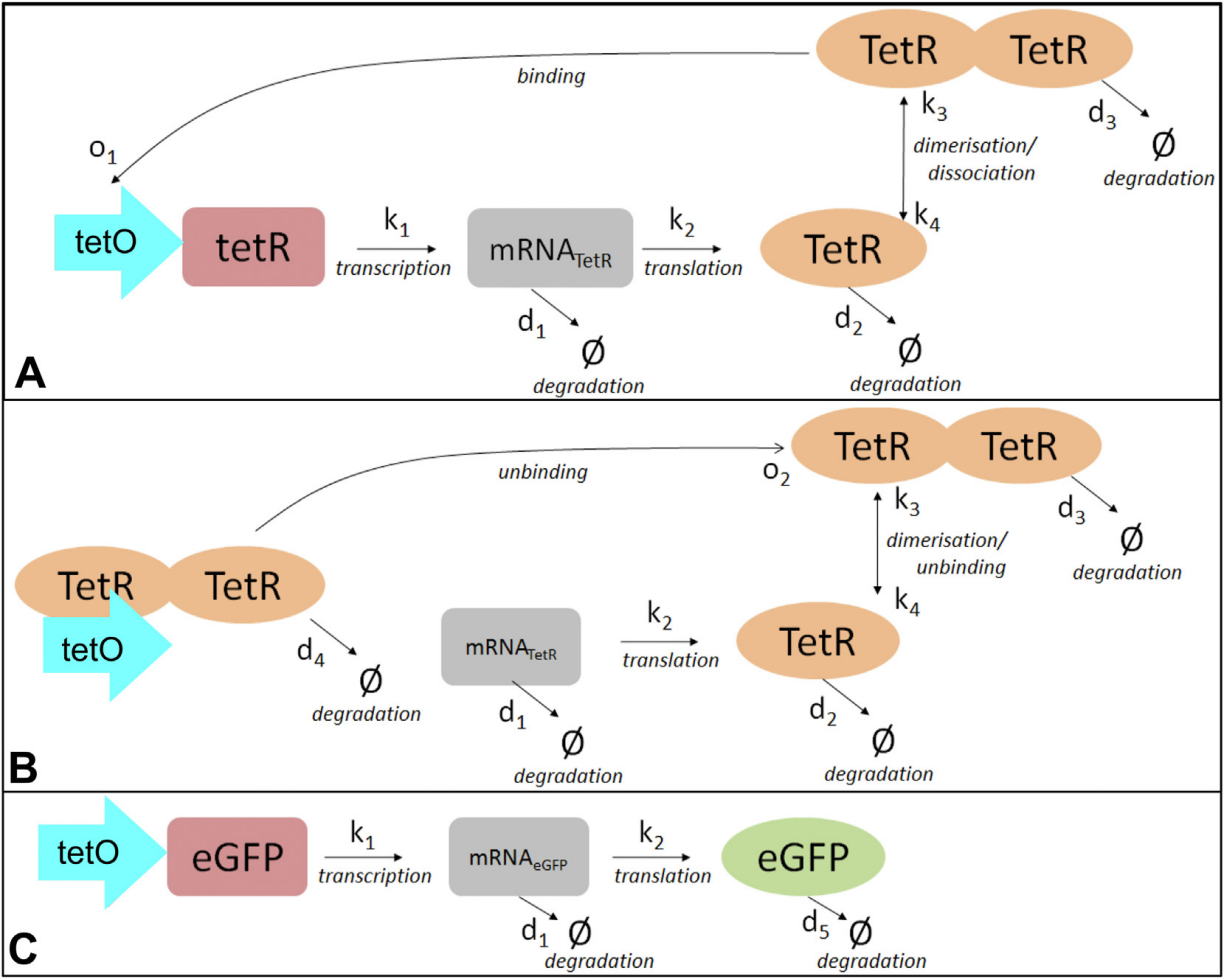
Overview of pYB-Stable-encoded species and reactions simulated by *an initio* modelling. Schematic overview of the seven molecular species (coloured shapes) defined in Table 2 and eleven reaction rates (arrows) defined in Table 4.

We simulated how TetR and Ub-eGFP expression would vary over time by *ab initio* modelling of the levels of seven molecular species (Figure 2) deemed most critical to the process (Table 2). We assumed that 14 reactions (Table 3) would determine the abundance of the 7 molecular species at any point in time and that the level of activity of these 14 reactions would be determined by 11 rate parameters (Table 4 and Figure 2). Values for protein translation stability and binding were derived from a Tigges et al. (2010) study with a mammalian cell oscillator, except for the degradation rate of Ub-eGFP which was determined directly (Borg 2015), and transcription data sourced from Stricker et al. (2008). Effects of the UbeK degron were not modelled explicitly but factored into the TetR dimer degradation rate.

**Table 2:** Species components encoded by the plasmid pYB-Stable.

	Species	Description	Equation(s)
1	mRNA_TetR_	The mRNA transcript arising from transcription of the tetR ORF	1.2
2	TetR	The protein arising from translation of mRNA_TetR_	1.3
3	TetR_2_	Homodimerised TetR protein	1.4
4	tetO	DNA sequence with high affinity for TetR dimer	1.5
5	tetO:TetR_2_	Complex formed by TetR_2_ binding to tetO	1.6
6	mRNA_Ub-eGFP_	The mRNA transcript arising from transcription of the Ub-eGFP ORF	1.7
7	Ub-eGFP	The protein arising from translation of mRNA_Ub-eGFP_	1.8

**Species components encoded by the plasmid pYB-Stable.** The species/components encoded by the plasmid pYB-Stable predicted to be critical for onset of Goodwin oscillation. Figure 2 provides an overview diagram of the molecular species and rates.

**Table 3:** Biochemical reactions chosen for *ab initio* modelling.

Reaction	Description
$R_{1}\;0\;\overset{k_{1}}{\to }\;mRNA_{tetR}$	Transcription
$R_{2}\;mRNA_{tetR}\;\overset{k_{2}}{\to }\;mRNA_{tetR}+TetR$	Translation
$R_{3}\;TetR+TetR\;\overset{k_{3}}{\to }\;TetR_{2}$	Dimerization
$R_{4}\;TetR_{2}\;\overset{k_{4}}{\to }\;TetR+TetR$	Dissociation
$R_{5}\;tetO+TetR_{2}\;\overset{0_{1}}{\to }\;tetO.TetR_{2}$	Binding
$R_{6}\;tetO.TetR_{2}\;\overset{0_{2}}{\to }\;tetO+TetR_{2}$	Unbinding
$R_{7}\;mRNA_{tetR}\;\overset{d_{1}}{\to }0$	Degradation
$R_{8}\;TetR\;\overset{d_{2}}{\to }0$	Degradation
$R_{9}\;TetR_{2}\;\overset{d_{3}}{\to }0$	Degradation
$R_{10}\;tetO.TetR\;\overset{d_{4}}{\to }\;tetO$	Degradation
$R_{11}\;0\;\overset{k_{1}}{\to }mRNA_{eGFP}$	Transcription
$R_{12}\;mRNA_{eGFP}\;\overset{k_{2}}{\to }\;mRNA_{eGFP}+eGFP$	Translation
$R_{13}\;mRNA_{eGFP}\;\overset{d_{1}}{\to }\;0$	Degradation
$R_{14}\;eGFP\;\overset{d_{5}}{\to }\;0$	Degradation

**Biochemical reactions chosen for *ab initio* modelling.** The biochemical reactions chosen for *ab initio* modelling of the dynamics of a putative Goodwin oscillator encoded by the plasmid pYB-Stable. The species are as described in Table 2 reaction rates *k*_i_, *o*_*i*_ and *d*_*i*_ are as described in Table 4.

**Table 4:** Rate parameters and distributions.

RATE	DESCRIPTION	DISTRIBUTION
*k*_*1*_	Rate of transcription of tetR and eFGP ORFs	*U (0.*18 min^−1^*, .*054 mol^−1^ min^−1^*)*
*k*_*2*_	Rate of translation of mRNA_tetR_ and mRNA_eGFP_	*U (0.*01 min^−1^*, 0.*03 min^−1^*)*
*k*_*3*_	Rate of dimerization of tetR	*U (0.*007 mol^−1^ min^−1^*, 0.*02 mol^−1^ min^−1^*)*
*k*_*4*_	Rate of dissociation of tetR_2_	*U (0.*0005 min^−1^*, 0.*0015 min^−1^*)*
*o*_*1*_	Binding rate of tetR_2_ to tetO	*U (0.*009 min^−1^*, 0.*027 mol^−1^ min^−1^*)*
*o*_*2*_	Unbinding rate of tetR_2_ from tetO	*U (0.*00005 min^−1^*, 0.*00015 min^−1^*)*
*d*_*1*_	Degradation rate of mRNA_tetR_ and mRNA_eGFP_	*U (0.*008 min^−1^*, 0.*026 min^−1^*)*
*d*_*2*_	Degradation rate of tetR	*U (0.*011 min^−1^*, 0.*035 min^−1^*)*
*d*_*3*_	Degradation rate of tetR_2_	*U (0.*011 min^−1^*, 0.*035 min^−1^*)*
*d*_*4*_	Degradation rate of tetR_2_ when bound to tetO	*U (0.*011 min^−1^*, 0.*035 min^−1^*)*
*d*_*5*_	Degradation rate of eGFP	*0.*004 min^−1^

Rate parameters and distributions. Biochemical reaction rate parameters and distributions used in *ab initio* modelling of the dynamics of the putative Goodwin oscillator encoded by the plasmid pYB-Stable. All reactions are provided in Table 3 and values in this table are based upon our observations of the Ub-eGFP half-life and an average transcription rate reported by Stricker et al. (2008), with all other values based on data reported by Tigges et al. (2010). Abbreviation U = units, mol = molecules, min = minutes. Figure 2 provides an overview diagram of the molecular species and rates.

The 7 molecular species (Table 2) and 14 reactions (Table 3) were described as a set of ordinary differential equations (ODEs) to predict how interplay of the biochemical reactions determined the changing abundance of each species changed over time. These ODEs were a deterministic representation of the network so to better reflect the stochastic nature of biological systems they were coded in SBML to enable their transformation into the more realistic chemical master equation of the Gillespie algorithm (1976). The Gillespie algorithm is a stochastic simulation algorithm (SSA), which uses probability measures to map out changes in species’ molecule numbers due to the biochemical reactions that occur over time, and bases the decision of when and which biochemical reaction next takes place on a probability measure. This feature is commonly used to introduce an element of randomness into gene network simulations (Elowitz and Leibler 2000; Stricker et al., 2008) to reflect the stochastic nature of the biological setting of the oscillation. The seven molecular species were represented as [x] in (1.2), (1.3), (1.4), (1.5), (1.6), (1.7), (1.8).

(1.2) $$\frac{d\left[mRNA_{tetR}\right]}{dt}=k_{1}\frac{\left[tetO\right]}{S+\left[tetO\right]}-d_{1}\left[mRNA_{tetR}\right]$$

(1.3) $$\frac{d\left[TetR\right]}{dt}=k_{2}\left[mRNA_{tetR}\right]-2k_{3}\left[TetR\right]\frac{\left[TetR-1\right]}{2}+2k_{4}\left[TetR_{2}\right]-d_{2}\left[TetR\right]$$

(1.4) $$\frac{d\left[TetR_{2}\right]}{dt}=k_{3}\left[TetR\right]\frac{\left[TetR-1\right]}{2}-k_{4}\left[TetR_{2}\right]-d_{3}\left[TetR_{2}\right]-o_{1}\left[TetR_{2}\right]\left[tetO\right]+o_{2}\left[TetR2.tetO\right]$$

(1.5) $$\frac{d\left[tetO\right]}{dt}=-o_{1}\left[TetR_{2}\right]\left[tetO\right]+o_{2}\left[TetR2.tetO\right]+d_{4}\left[TetR_{2}.tetO\right]$$

(1.6) $$\frac{d\left[TetR_{2}.tetO\right]}{dt}=o_{1}\left[TetR_{2}\right]\left[tetO\right]-o_{2}\left[TetR2.tetO\right]-d_{4}\left[TetR_{2}.tetO\right]$$

(1.7) $$\frac{d\left[mRNA_{Ub-eGFP}\right]}{dt}=k_{1}\frac{\left[tetO\right]}{S+\left[tetO\right]}-d_{1}\left[mRNA_{Ub-eGFP}\right]$$

(1.8) $$\frac{d\left[Ub-eGFP\right]}{dt}=k_{2}\left[mRNA_{Ub-eGFP}\right]-d_{5}\left[Ub-eGFP\right]$$

All reactions, except for dimerisation and transcription, were modelled as mass action laws. Dimerisation took into account the multiple possible routes to TetR dimer-based repression (Gillespie 1976). Transcription was modelled as a Hill function (Elowitz and Leibler 2000; Fung et al., 2005; Tigges et al., 2009; Sheinman and Kafri 2012) and assumed a gene dosage of one copy per cell. By relating transcription directly to whether tetO was bound or not, rather than the abundance of TetR dimers, a direct estimation of ongoing transcription was generated.

Combinations of reaction activity levels were randomly selected via the ABC Sysbio software (Table 1) and input into the Direct Method Gillespie algorithm (Gillespie, 1977) to generate a time series of the changing quantities of different molecules. Gillespie time series simulations were set to model component dynamics over a time-span of 1000 min. The quantity of each species was recorded at 0.5 min intervals. 500 simulations were carried out, implying 500 different parameter combinations were tested based on simple random sampling. Carrying out an exhaustive scan of the parameter space would have been infeasible since there are over 4×10^100^ possible parameter combinations based on the above uniform distributions and the power of the ABC-Sysbio program. The set of simulations was considered to represent a population of 500 cells rather than just one cell sampled for 500 times.

**Figure 3:**
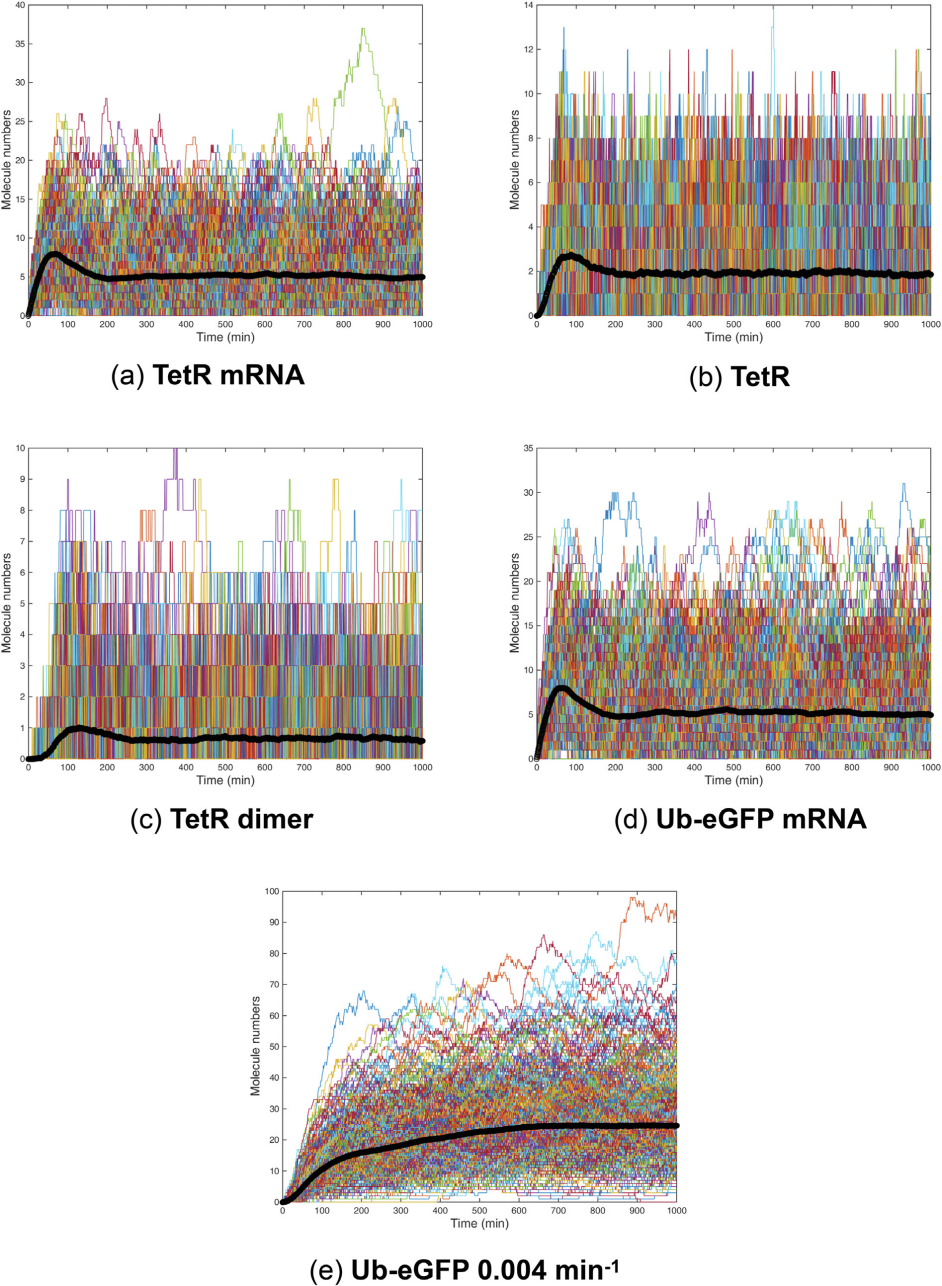
500 Gillespie-simulated trajectories of pYB-Stable-encoded species over 1000 min. Gillespie simulations were plotted of the number of molecules of (a) mRNA_TetR_, (b) TetR protein, (c) TetR_2_ homodimer, (d) mRNA_Ub-eGFP_ and (e) Ub-eGFP protein expressed from pYB-Stable over time. Black line data set in each graph is the average number of molecules of the indicated molecular species. Starting values for all species were set to zero except the tetO DNA sequence (not plotted), which was set to one.

**Figure 4:**
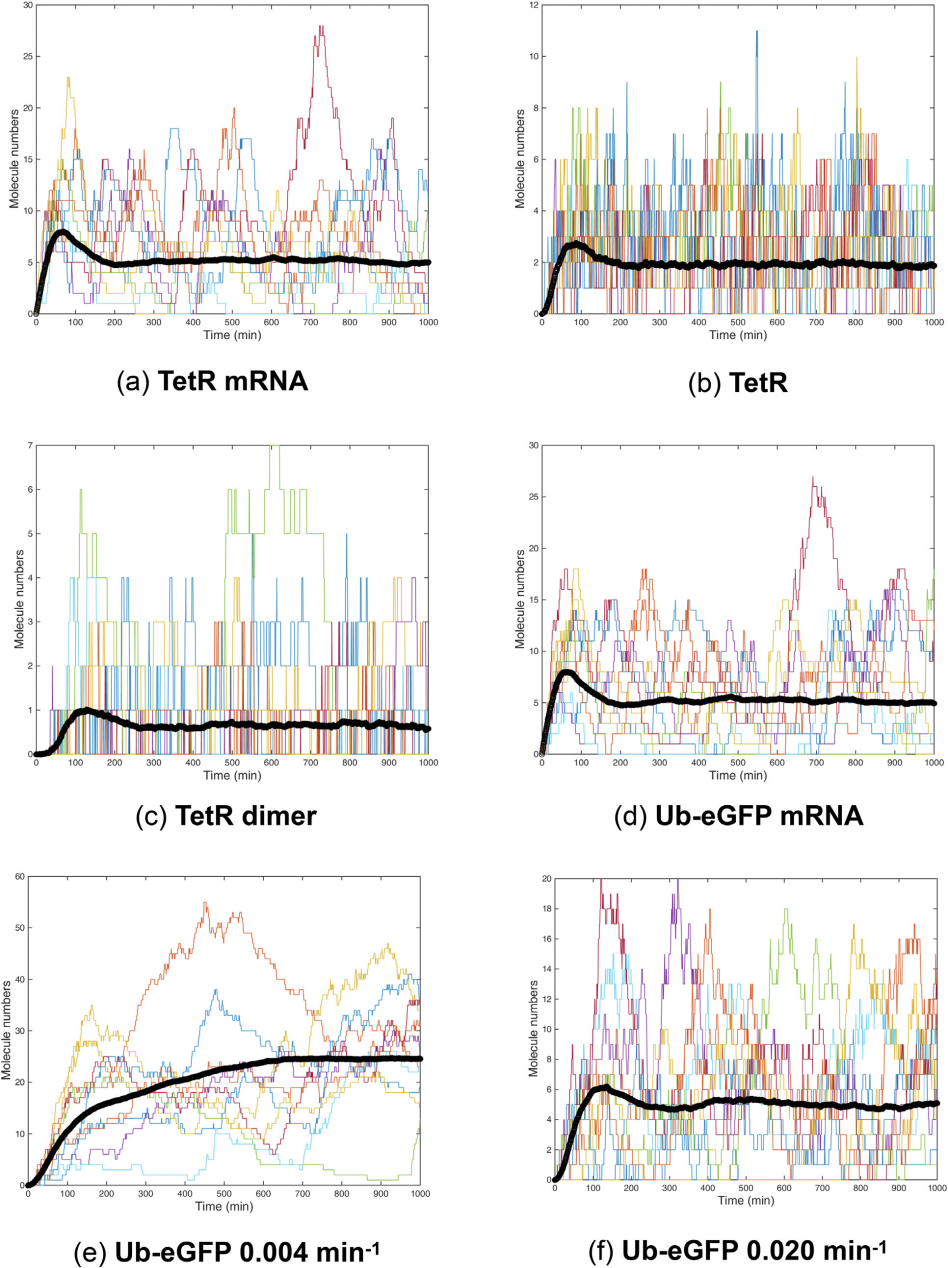
Ten Gillespie-simulated trajectories of pYB-Stable-encoded species over 1000 min. Time series trajectories arising from ten *ab initio* Gillespie simulations selected at random from the 500 plotted in Figure 3. Each panel shows Gillespie simulations of a putative Goodwin oscillator encoded by pYB-Stable over time. Number of molecules over time plotted for 10 simulations of (a) mRNA_TetR_, (b) TetR protein, (c) TetR_2_ homodimer, (d) mRNA_Ub-eGFP_, (e) Ub-eGFP protein with decay rate set at 0.004 min^−1^ and (f) Ub-eGFP protein with decay rate set at 0.020 min^−1^. Black line data set in each graph is the average number of molecules of the indicated molecular species. Starting values for all species were set to zero except the tetO DNA sequence (not plotted), which was set to one.

Figure 3 shows the 500 time series trajectories for TetR mRNA, TetR protein, TetR protein dimer, Ub-eGFP mRNA and Ub-eGFP protein, with starting molecule numbers set at 0. Time series trajectories for vacant tetO and TetR-bound tetO were not plotted as they simply switch between binary states of 0 (0% bound) and 1 (100% bound). For clarity, 10 randomly selected simulations from Figure 3 were also plotted alone in Figure 4. The simulations plotted in Figures 3 and 4 predicted that all molecular species involve in TetR and Ub-eGFP expression would oscillate continually. Protein dynamics of the same species were not synchronised over the different simulations, except for a calibration period at the beginning of the simulation, as reflected in the average time-series (black band in Figures 3 and 4). Simulated TetR and Ub-eGFP mRNA levels showed similar oscillation profiles whereas TetR protein and TetR protein dimer oscillation had a lower amplitude and shorter period than Ub-eGFP protein levels. Ub-eGFP showed a trend of increasing sinusoidal oscillations (Figure 4E and 4.F), similar to observations for GFP oscillation reported by Elowitz and Leibler (2000). Simulations performed using a higher Ub-eGFP degradation rate of 0.020 min^−1^ (Figure 4E), or the experimentally determined (Borg 2015) degradation rate of 0.004 min-^1^ (Figure 4F), showed a more uniform sinusoidal oscillation pattern with lower amplitudes.

**Figure 5:**
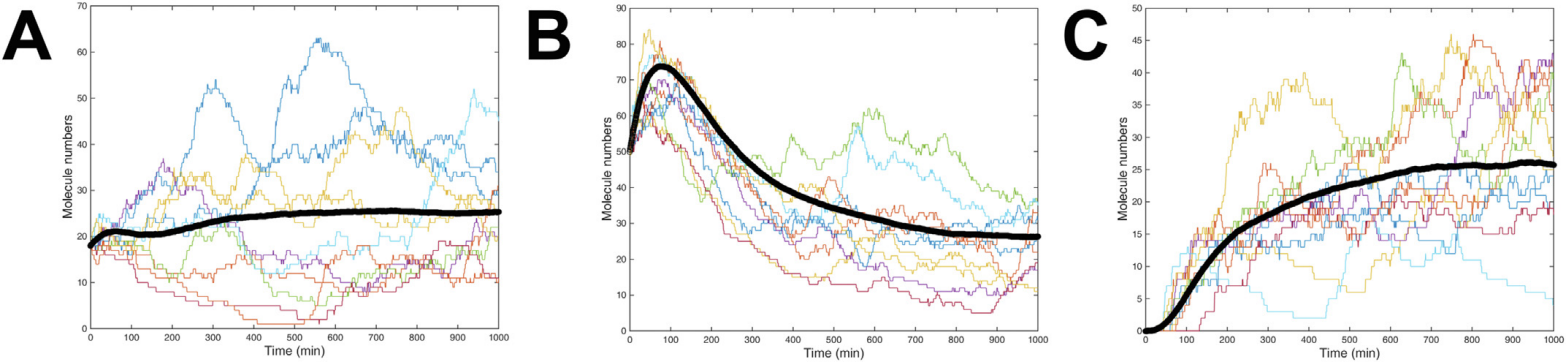
Ten Gillespie-simulated trajectories of pYB-Stable-encoded Ub-eGFP over 1000 min. Plot of Ub-eGFP trajectories arising from 10 *ab initio* Gillespie simulations, selected at random from 500, over time, when: A) starting numbers of molecules were set at 1 for tetO, zero for tetO bound to TetR_2_ homodimer and randomly at values between 1 and 20 for mRNA_TetR_, TetR protein and mRNA_Ub-eGFP_, B) set at 1 for tetO, zero for tetO bound to TetR_2_ homodimer and 50 for all other species, and C) set at one for tetO bound to TetR_2_ homodimer and zero for all other species. Black line data set in each graph is the average number of molecules of the indicated molecular species.

Ideally the emergence of Ub-eGFP oscillation would be robust to wide variations in the initial levels of activity, immediately following its genomic integration and the first transcription, translation, TetR dimerisation and tetO binding events. To predict if oscillation of Ub-eGFP would be independent of starting conditions, sets of 500 Gillespie simulations were performed with three different starting conditions and 10 randomly selected simulations for each plotted for graphical clarity (Figure 5).

For the simulations plotted in Figure 5A and 5B, the vacant tetO was set to 100% and TetR dimer-bound tetO set to 0%. Starting quantities of TetR mRNA, TetR protein, TetR protein dimer, Ub-eGFP mRNA and eGFP protein were each then set to a random number between 1 and 20 (Figure 5A) or between 1 and 50 (Figure 5B). For the simulation plotted in Figure 5C, TetR dimer-bound tetO was set to 100% and a starting quantity of zero was set for TetR mRNA, TetR protein, TetR protein dimer, Ub-eGFP mRNA and eGFP protein. Oscillation arose in all three simulations, which encouraged us to move forward with building and *in vivo* testing a pYB-Stable plasmid to encode the oscillatory gene network.

### 6.3 Designing and building a plasmid encoding a trypanosomal Goodwin oscillator gene network

**Figure 6:**
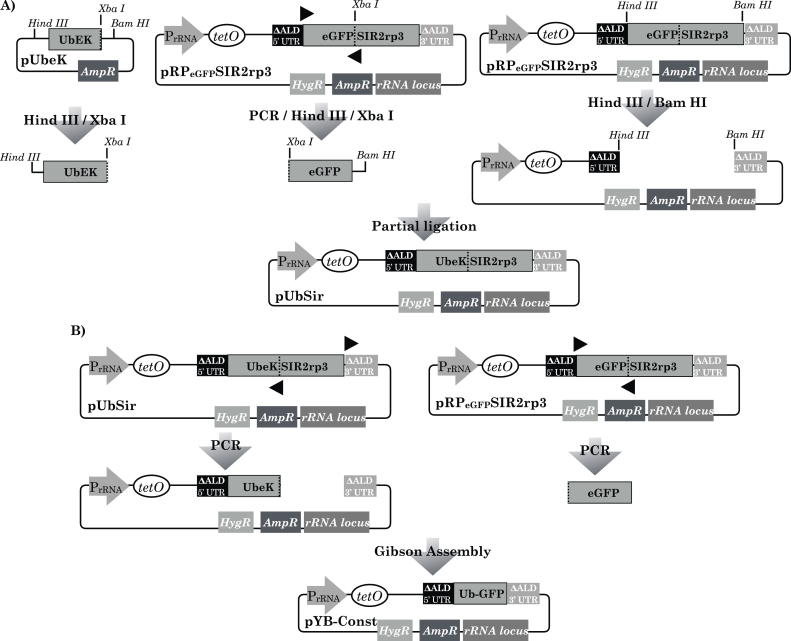
Design and assembly of pYB-Const plasmid encoding a gene intended to be non-oscillating. A) A three-fragment ligation was designed for fragments generated from the plasmids pUbeK and pRPeGFPSIR2rp3. The P_rRNA_ promoter (grey arrow) and untranslated regions (UTR) present in pRPeGFPSIR2rp3 are described in the Results and Discussion section. An ampicillin (Amp^R^) gene enabled plasmid propagation in *E. coli*, a segment of the *T. b. brucei* rRNA locus (box labelled rRNA locus) featuring a lone *Not I* site enabled targeted integration into the *T. b. brucei* genome and a hygromycin (Hyg^R^) gene enabled selection of stable T. b. brucei transfectants. The leftmost plasmid diagram is of pUbeK, a plasmid based on a pUC57 backbone and encoding an N-terminal ubiquitin-eK degron (UbeK) partial reading frame, flanked by an upstream *Hind III* site and a downstream *Bam HI* site, with an in-frame *Xba I* site spanning the end of the protein-coding region (dashed line). The top, middle plasmid diagram is of pRPeGFPSIR2rp3. Mutagenic PCR with pRPeGFPSIR2rp3 as template, with forward primer (black triangle pointing right), and reverse primer: (black triangle pointing left), amplified a fragment encoding a section of an enhanced green fluorescent protein (eGFP) ORF. Digestions with *Hind III* and *Xba I* were used to isolate an UbEK fragment from pUbeK and a backbone fragment from pRPeGFPSIR2rp3 (rightmost plasmid diagram). All three-fragment ligation attempts failed and resulted in the unintended plasmid product, pUbSir, encoding an UbeK-SIR2rp3 fusion protein (lowermost plasmid diagram in panel). B) PCR was performed with pUbSir (plasmid diagram on the left) as template with primers (black triangle pointing right) and (black triangle pointing left) to amplify the plasmid without the SIR2rp3 region of the UbeK-SIR2rp3 ORF. PCR was also performed with pRPeGFPSIR2rp3 (plasmid diagram on the right) as template with forward primer (black triangle pointing right) plus reverse primer (black triangle pointing left) to generate a fragment encoding a partial eGFP ORF with no start codon and only a stop codon. Gibson assembly of the two PCR products yielded the pYB-Const plasmid (lowermost plasmid diagram).

We designed the plasmid, pYB-Const, to encode a control transgene intended to give rise to a constant level of GFP expression (Figure 6B, lowermost plasmid diagram). To favour a gene dosage of one insert per cell, a copy of a segment of the *T. b. brucei* encoding a non-transcribed ribosomal RNA spacer was used, containing a Not I restriction site for linearization followed by targeted integration of the plasmid by homologous recombination (Alsford et al., 2005; Alsford and Horn 2008). A hygromycin resistance gene under control of a constitutive promoter was also present in pYB-Const to select for stable transformants and in the reverse orientation to the GFP expression cassette to prevent any possibility of transcriptional read-through from that cassette (Alsford et al., 2005). The strong, constitutive trypanosomal PrRNA promoter (Alsford and Horn 2008) was used to drive GFP expression. A tetO sequence was present immediately downstream of the PrRNA promoter and should remain unbound due to the lack of a TetR gene elsewhere in pYB-Const and the *T. b. brucei* genome. To encode a destabilised version of enhanced green fluorescent protein we assembled a fusion of eGFP and a novel ‘UbeK’ degron. The UbeK degron consisted of 76 residues of native *T. b. brucei* ubiquitin protein, at the N-terminus and ending in a leucine residue to signal proteasomal degradation (Dantuma et al., 2000; Gonda et al., 1989), followed by a short ‘e^K^’ region which featured an additional proteasomal degradation signal of two lysine residues flanking an arginine (Johnson et al., 1995). We designed the Ub-eGFP ORF to be flanked (Clayton and Shapira 2007) by 5′ and 3’ ‘ALD’ untranslated regions (UTRs) to promote mRNA stability and strong levels of translation (Drozdz and Clayton 1999; Clayton 1999). Assembly of pYB-Const (see Materials and Methods for full details) was achieved via a first round of ligation (Figure 6A) which yielded an intermediate plasmid, pUbSir, which was then used in a second and final ligation to yield pYB-Const (Figure 6B).

**Figure 7:**
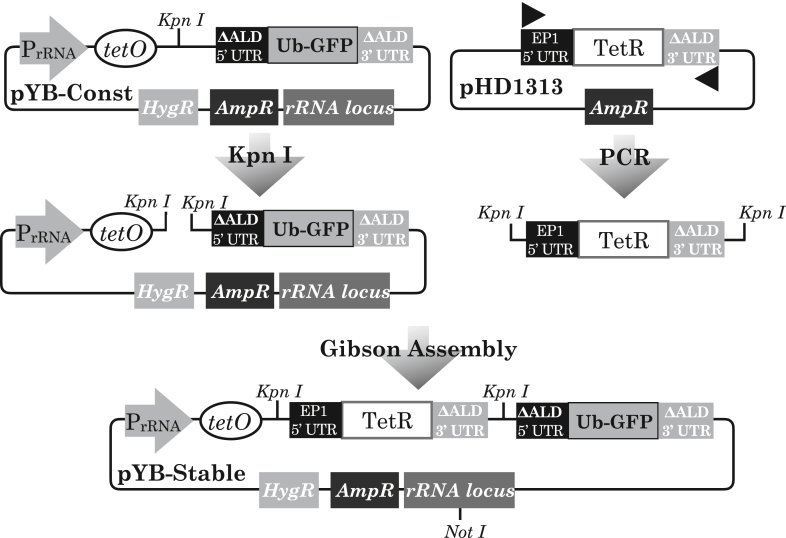
Design and assembly of pYB-Stable plasmid encoding a gene intended to oscillate. The pYB-Const plasmid (top left plasmid diagram) was linearised by digestion of a lone *Kpn I* site. PCR was performed with pHD1313 (top right plasmid diagram) as template with a forward primer (black triangle pointing right) plus reverse primer (black triangle pointing left) to generate a fragment encoding the TetR ORF with both primers including regions identical to the intended insertion site within the pYB-Const destination plasmid. Gibson assembly of the purified PCR product and linearised plasmid yielded the pYB-Stable plasmid.

A second plasmid, pYB-Stable, was designed to encode a gene network intended to give rise to stable oscillations of a detectable GFP signal. pYB-Stable featured the same genetic elements present in pYB-Const detailed above, with the addition of a tetR ORF between the tetO and Ub-eGFP ORF. The tetR ORF was flanked upstream and downstream respectively by the EP1-5’ (Schurch et al., 1997) and ALD-3’ UTRs, again to promote mRNA stability and translation. Restriction enzyme linearsation of pYB-Const and PCR of the plasmid pHD1313, to obtain the TetR ORF, were followed by Gibson assembly to generate the final pYB-Stable plasmid (Figure 7).

**Figure 8:**
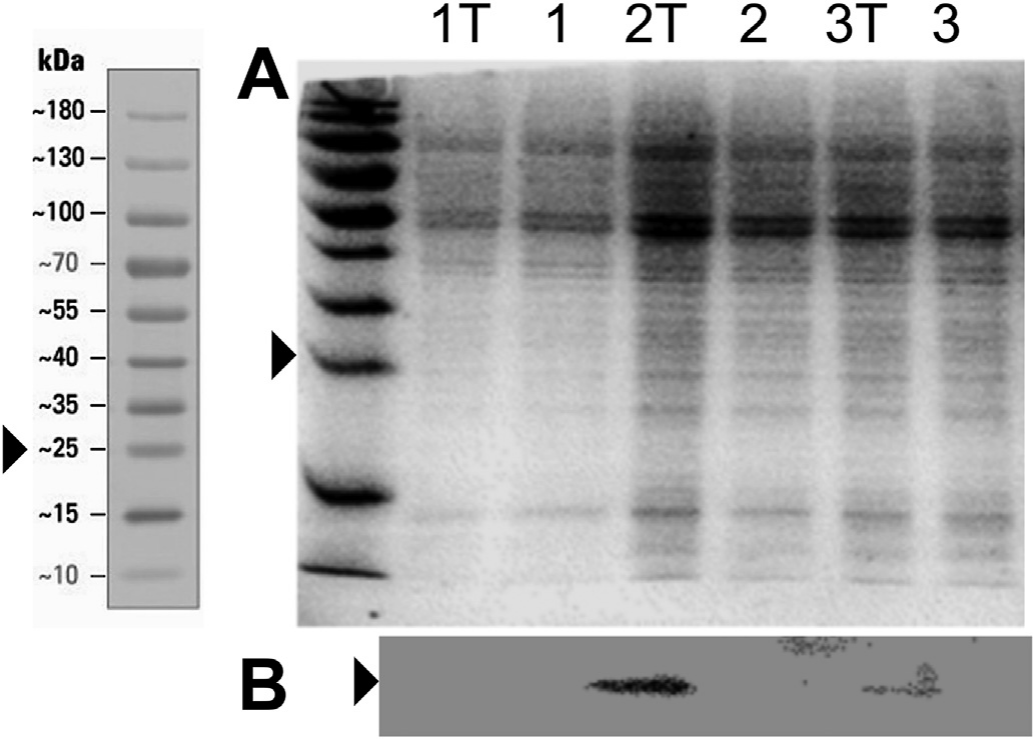
Coomassie and anti-GFP antibody staining of cell samples run on SDS PAGE. Cells from clonally derived populations stably transfected with pYB-Stable, TbGOS01 (1), TbGOS02 (2), and TbGOS03 (3), were analysed by SDS PAGE, with number label indicating cell line. The electrophoresed cell sampes were stained with Coomassie (Panel A) or with anti-GFP antibody after transfer to nitrocellulose (Panel B). Black arrows indicate migration distance travelled by a 25kDa protein of a commercial protein ladder (leftmost panel), in all three panels. Lanes labelled with ‘T’ in addition to number, indicates that cells were cultivated on the presence of 10 μg/mL tetracycline prior to electrophoresis.

Both pYB-Const ansd pYB-Stable were linearised by *Not I* digestion and used to stably transfect PCF *T. b. brucei* cells. Hygromycin was used to select a mixed population of transformants from which clonally derived populations were subsequently isolated. PCF cells stably transfected with pYB-Const, resulted in Ub-eGFP expression in the presence or absence of 1 μg/mL tetracycline (Borg 2015). Three clonally derived populations stably transfected with pYB-Stable were isolated and cultivated in the presence or absence of 10 μg/mL tetracycline and cells analysed by SDS PAGE with Coomassie staining (Figure 8A) and Western blotting with an anti-eGFP antibody (Figure 8B). Western blot analysis (Figure 8B) revealed that clonally derived populations TbGOS02 and TbGOS03 were both positive for Ub-eGFP expression only when tetracycline was present. This observation was consistent with the hypothesis that both the TetR and Ub-eGFP proteins, encoded by integrated pYB-Stable in these two clonally derived populations, were expressed and functional. Clonally derived population TbGOS02 was taken forward for subsequent experiments. The growth characteristics of TbGOS02 did not markedly diverge from that of the parent strain in the presence or absence of tetracycline (Borg 2015).

### 6.4 Establishing an experimental framework for following fluorescence in live *T. b. brucei* cells

Having built plasmids and transfectant cell lines based on our designs and simulations, we sought next to develope an experimental setup to test if our SGN directed oscillation of GFP levels in live *T. b. brucei* cells. To this end we established a setup to capture images of fluorescence in living trypanosomes (FILT) to obtain a series of fluorescent and phase contrast images of living toPCF *T. b. brucei* cells to follow levels of Ub-eGFP expression over time.

PCF *T. b. brucei* cells are highly motile in solution, exhibiting swim-and-tumble phases of movement and travelling at up to 5.6 μm/s (Weisse et al., 2012). Capturing images of individual cells therefore required a setup in which cell movement in x-, y- and z-planes was constrained while cell viability was preserved over as long period as possible. This requirement was key, as Kinetoplast cells tend to lose viability when immobilised (Price et al., 2010). Furthermore, the setup had to enable identification of individual cells over multiple image time points and favour suspension of single cells over aggregations of multiple cells.

**Figure 9:**
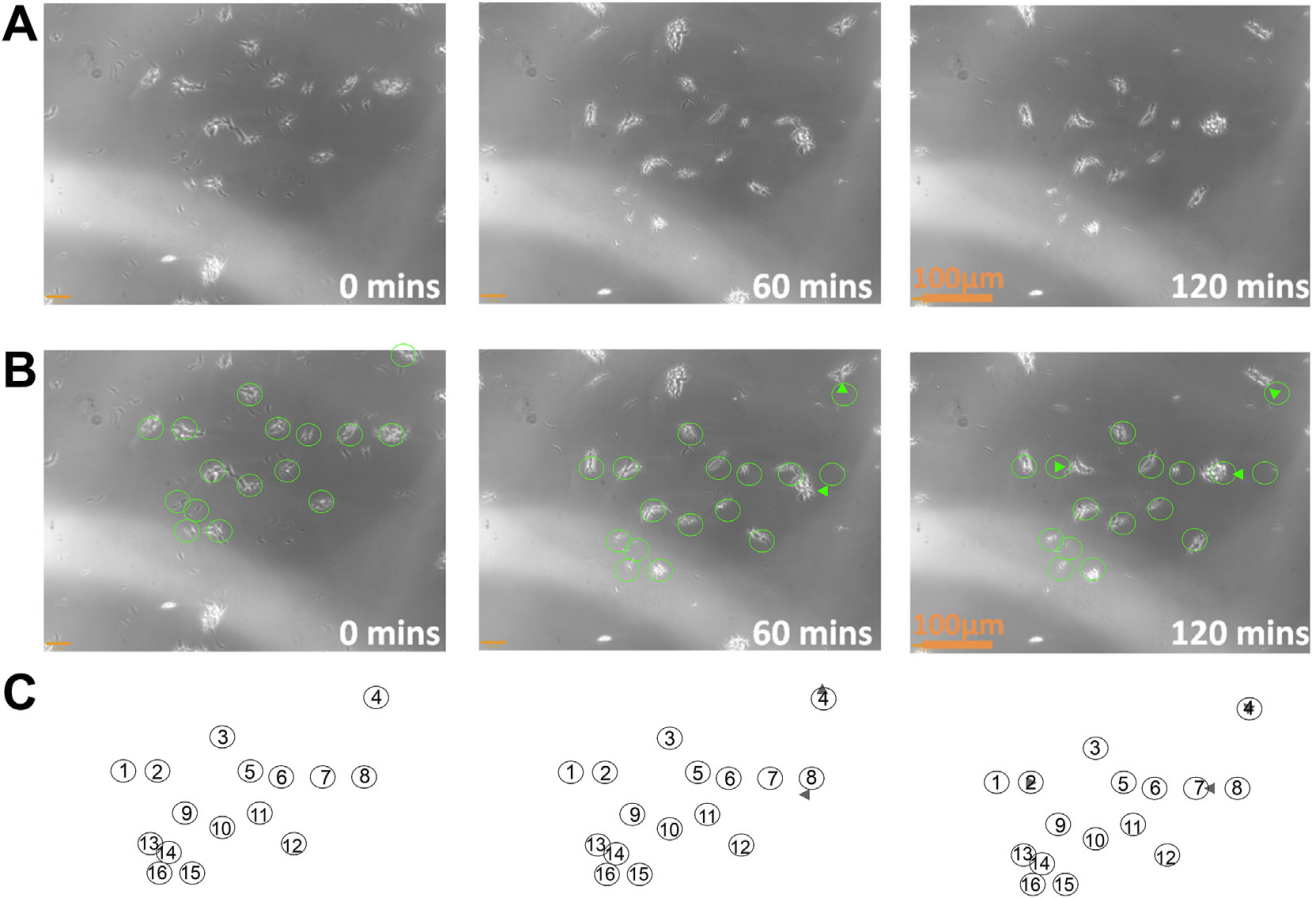
Immobilisation of live cells over a 120-minute time period. Phase contrast images and analysis of cells resuspended in 40% w/v Cygel™ solution within the FILT framework as detailed in Materials and Methods. Row A) Images captured after zero (0 min), 60 (60 min) and 120 (120 min) minutes. Row B) Green circles were drawn over the 16 cells within the image field for the zero minutes image. The same collection of circles was copied and pasted *en bloc* onto the 60 min and 120-minute images. Green triangles were inserted into the 60 min and 120-minute images to indicate where cells had moved position relative to the circles in the zero-minute image. Row C) Green circles of the zero-minute image have been re-coloured to black, enumerated and had the background image deleted. This pattern of enumerated circles was pasted beneath the 60 min and 120-minute images and the green triangles from row B) re-coloured to dark grey for clarity.

Cygel™ is a solid, transparent gel at room temperature and a liquid when cooled below this temperature. Price et al. (2010) used Cygel™ solutions to immobilise PCF *T. b. brucei* for up to 3 h. In this study we defined viability as the retention of the ability to show undulatory movement in a fixed position. At least 50% of cells suspended in 40% w/v solutions of Cygel™ in SDM 79 growth media retained viability for 4 h outside of an incubator in a volume of 4 μL within a 5 mm 12-well slide placed within an AxioPlan2 microscope as described in Materials and Methods. Semi-quantitative data (Figure 9) was gathered regarding cell movement within this setup over time. Figure 9 shows data from a representative experiment in which 13 cells embedded in 40% w/v solutions of Cygel™ (81.4%), from a selection of 16 individual cells in the field of view, retained their position over 120 min. The FILT experimental setup was therefore taken forward to capture fluorescence data from the cells of the clonally derived TbGOS02 strain.

### 6.5 Measuring fluorescence over time in cells harbouring a putative Goodwin oscillator

The Goodwin oscillator encoded in pYB-Stable (Figure 1A) is in theory tuneable by altering tetracycline concentration. At zero tetracycline concentration TetR-based repression of PrRNA promoter, by binding tetO, would be maximal and would decrease only in line with the half-life of TetR, which contains no degron. As such, in the absence of tetracycline, Ub-eGFP expression from pYB-Stable would be predicted to oscillate with maximum duration between periods of expression, a situation in which Ub-eGFP detection may be difficult to capture by taking images of live cells, and certainly would average a low level of signal by Western blotting. As anticipated, no cellular fluorescence was detectable when imaging of live cells in the absence of tetracycline (Borg 2015). Figure 8B also shows no Ub-eGFP detection by Western blot of TbGOS02 cells grown in the absence of tetracycline and a strong Ub-eGFP band for cells grown in 10 μg/mL tetracycline.

At less than maximal and greater than zero tetracycline concentrations, we predicted that tetracycline would in effect act to modulate the functional half-life of tetR dimer binding to tetO, because tetO binding/unbinding events are mediated by tetracycline binding to tetR dimer. Given the effects of zero and maximal tetracycline were as predicted (Figure 8B), we anticipated that a critical range of tetracycline concentration would favour oscillations of cellular fluorescence that could be measured within the FILT experimental setup. Sampling a selection of tetracycline concentrations, decreasing in increments from 10 μg/mL, we identified 10 fg/mL as the lowest tetracycline concentration that resulted in observable fluorescence within TbGOS02 cells in the FILT setup. As cells were cultivated at a concentration of 1×10^6^ cells/mL, this gives an average of 135 tetracycline molecules/cell (tetracycline molecular mass = 444.435 Da).

**Figure 10:**
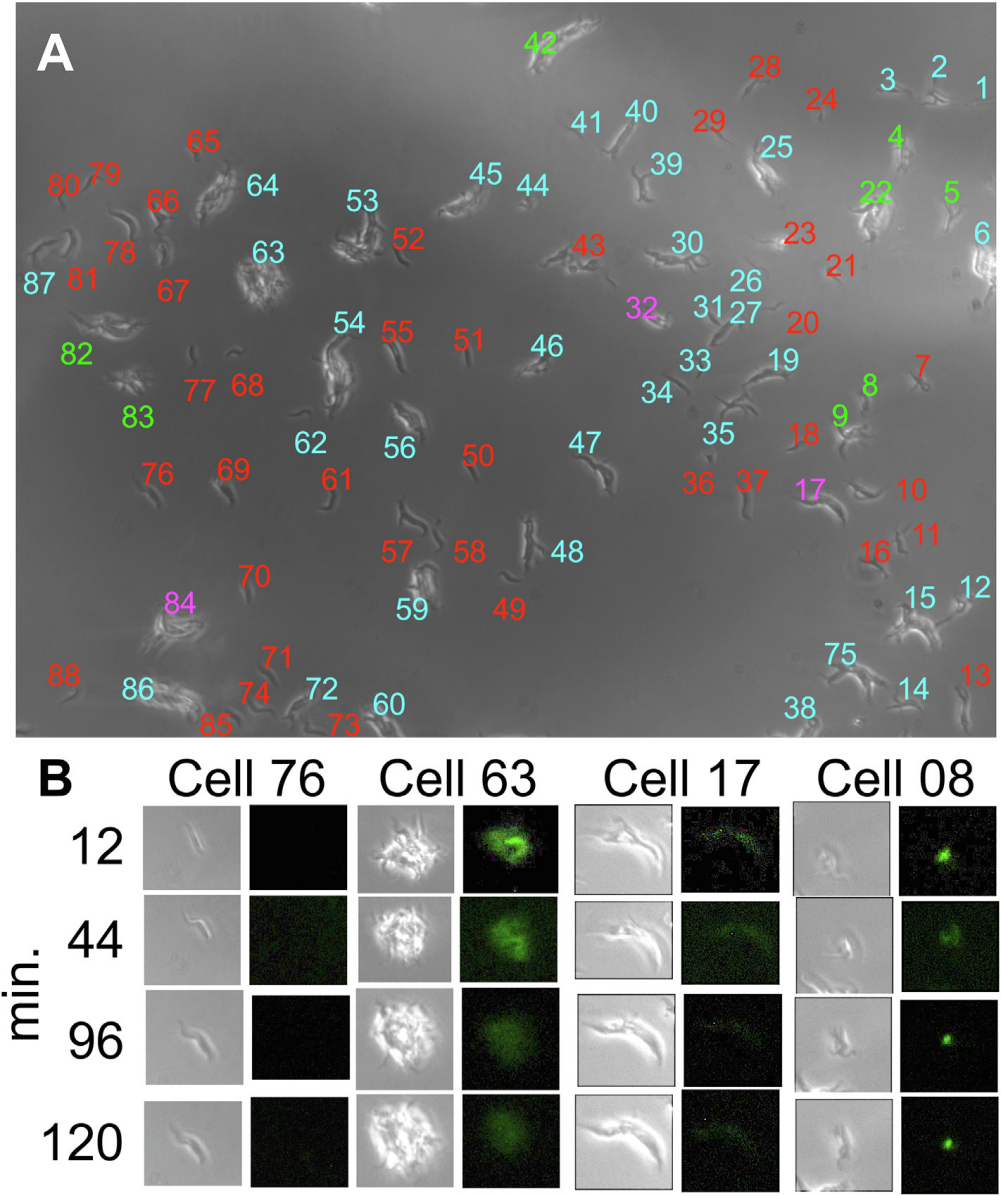
Fluorescent image signal in cells harbouring pYB-Stable over a 120-minute period. Cells of clonally derived population TbGOS02 were cultivated for 24 h in 10 fg/mL tetracycline before being resuspended in 40% w/v Cygel™ solution within the FILT framework, as detailed in Materials and Methods. Phase contrast and fluorescent images were captured every 2 min for 120 min. All images are available in Borg et al. (2021b). A) Phase contrast image captured after 2 min in the FILT setup in which 88 cells/groups of cells were enumerated. Analysis of all 60 consecutive images revealed four scenarios: 40 of the cells (red numbers) showed no fluorescent signal, 37 (blue numbers) gave a fluorescent signal that decreased over time, 3 cells (purple numbers) gave a fluorescent signal that remained constant over time and 8 cells (green numbers) showed cycles of increase, decrease and increase in fluorescent signal over time (9.1% of total cells in the image field). B) Panel of images of exemplar cells, taken at indicated time points, to illustrate the four scenarios observed across the image set. Cell 76 showed no fluorescent signal, cell 63 gave a fluorescent signal that decreased over time, cell 17 gave a fluorescent signal that remained constant over time and cell 08 showed cycles of increase, decrease and increase in fluorescent signal over time.

TbGOS02 cells were cultivated for 24 h in the presence of 10 fg/mL tetracycline, transferred to the FILT setup and phase and fluorescent images captured every 2 min for 120 min. Examination of 60 phase contrast and 60 fluorescent image files, all of which are available at Borg et al. (2021b) revealed that each image featured 88 cells (Figure 10A), all of which were present and could be enumerated for all images. Each cell was assigned a number from 1 to 88. The 60 images showed fluorescence increased, decreased and increased again in 8 cells (9.1% of total), remained constant in 3.4%, reduced gradually over the 120 min for 42% of cells and was absent from 45.5% of cells. Sample images of cells exhibiting each of these four patterns of fluorescence; cells 08, 17, 63 and 76 respectively, are provided in Figure 10B. The rise, fall and rise of fluorescence observed in 9.1% of cells in the captured image set were consistent with the ability of expression cassette encoded in pYB-Stable to function as a genetic oscillator.

### 6.6 Quantitative analysis of fluorescence in selected cells harbouring a putative Goodwin oscillator

**Figure 11:**
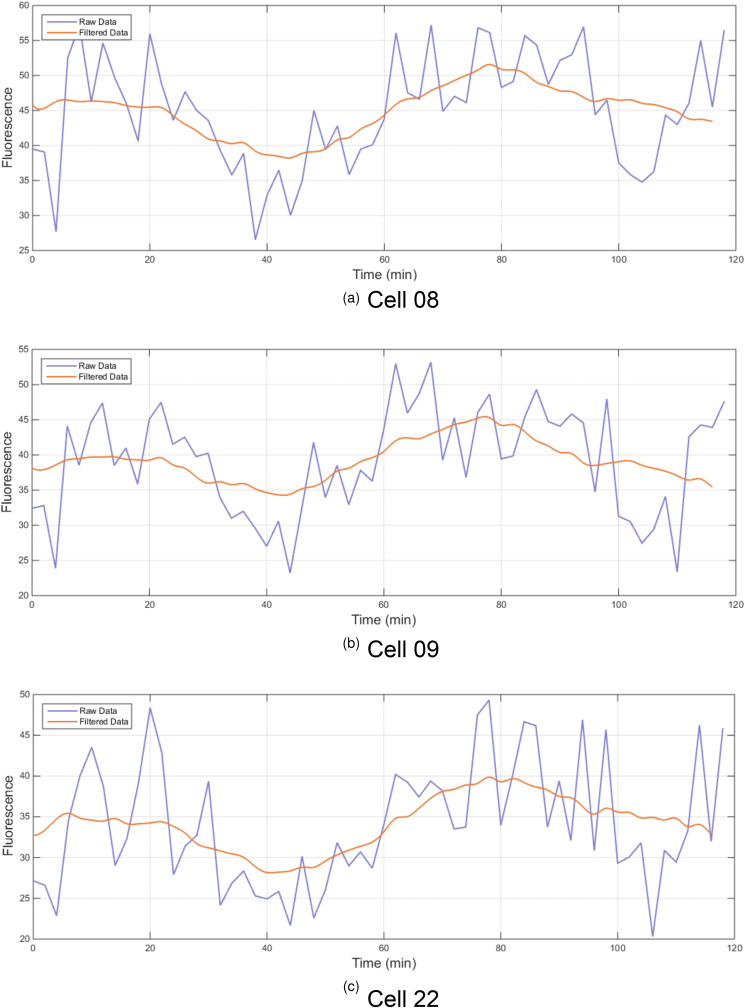
Raw and noise-filtered fluorescence data from still images of three TbGOS02 cells. Raw quantified fluorescence data (blue data set, arbitrary units) and imputed, noise-filtered and smoothed data (orange) for cell 08, (A), 09 (B) and 22 (C), using cell numbering system from Figure 9.

The 8 oscillating cells were ranked with respect the extent to which they did not form clumps with other cells and remained within the plane of vision. The three best cells by these criteria, cells 08, 09 and 22, were taken forward for quantitative image analysis, starting with background correction and automated tracking. For each of the three cells, only one of the 60 data points was set aside due to the cell falling outside of the plane of focus. For the missing data point an average of the value of the three previous and three subsequent data points was used to provide an imputed data point. The overall data set was then noise-filtered to extract the underlying trend from the stochastic environment. Plotting the raw quantified fluorescence data (Figure 11) showed an oscillating trend for all three cells.

**Figure 12:**
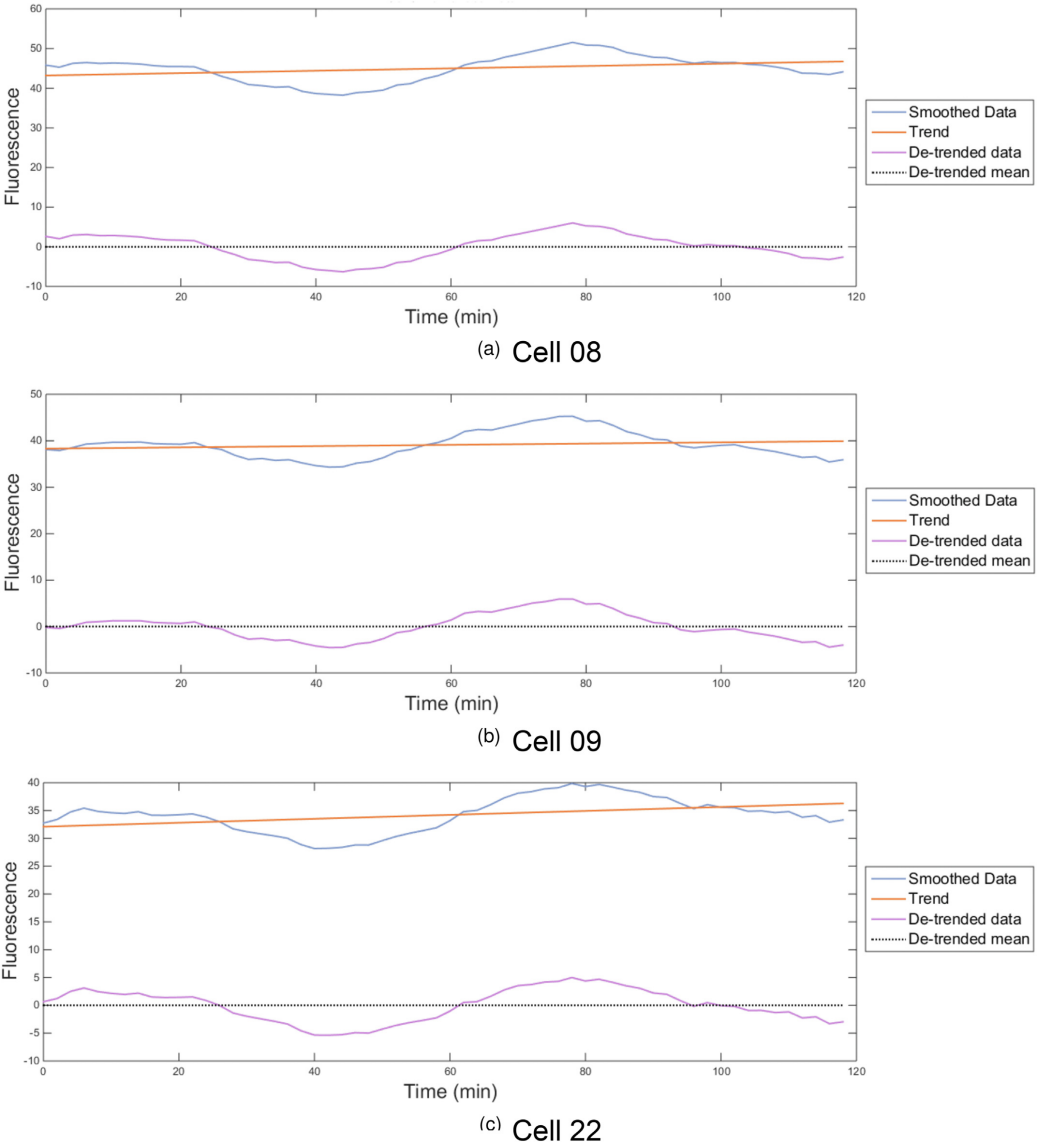
Smoothed and de-trended fluorescence data from images of three TbGOS02 cells. Imputed, noise-filtered and smoothed fluorescence data plotted in Figure 11 was smoothed and de-trended as described in Materials and Methods. For cell 08, (A), 09 (B) and 22 (C), imputed, noise-filtered, smoothed fluorescence data (arbitrary units) was plotted (blue), the linear trend of this data (orange) to obtain the detrended data (purple) alongside the de-trended mean (black).

Moving average filters were previously used by Tigges et al. (2009) to smooth oscillations and eliminate the effects of noise in fluorescence data from cells harbouring synthetic gene oscillators. Here we used a moving average filter (Oppenheim et al., 1999) with a window in which the average value of each data point was derived from that averaged point plus eight time points before and after it. The data set was further processed using cubic spline interpolation (Yang et al., 2013; Chen et al., 2009; Morrissey et al., 2011), whereby polynomials were fitted to sections of the noise-filtered data to smooth connections. The imputed, noise-filtered and smoothed functions for the three cells (Figure 12) show a sinusoidal trajectory, having a period of 50 min and a varying amplitude with a linear growth trend. These data were consistent with protein expression from the pYB-Stable plasmid being oscillatory, with hourly oscillations.

Fung et al. (2005), Gao et al. (2010) and Li et al. (2012) previously used phase space analysis to quantitate the oscillatory behaviour of synthetic gene networks. Phase space analysis enables all the possible states (values) of a selected variable within a defined network to be represented. Here we elected to reconstruct the phase space for the data captured from cells 08, 09 and 22. However, phase space analyses require true ideal oscillatory data sets, in which the amplitude of peaks and drops in protein expression remain constant. The smoothed fluorescence data set obtained for (Figure 12, blue line) showed an increasing average linear trend. We attributed this to net accumulation of Ub-eGFP over each round of oscillation, as the number of Ub-eGFP molecules was not degraded to zero prior to the next round of protein expression. To de-trend these data we applied the following statistical processing steps. Firstly, the mean value of the trajectory was subtracted from each data point of the smoothed dataset. A linear trend line (Figure 12 – orange trend line) was fitted to this smoothed dataset, with a mean of 0 (Figure 12 – black dotted trend line). The trend was then removed by subtracting the linear growth to obtain de-trended oscillations (Figure 12 – purple trend line).

**Figure 13:**
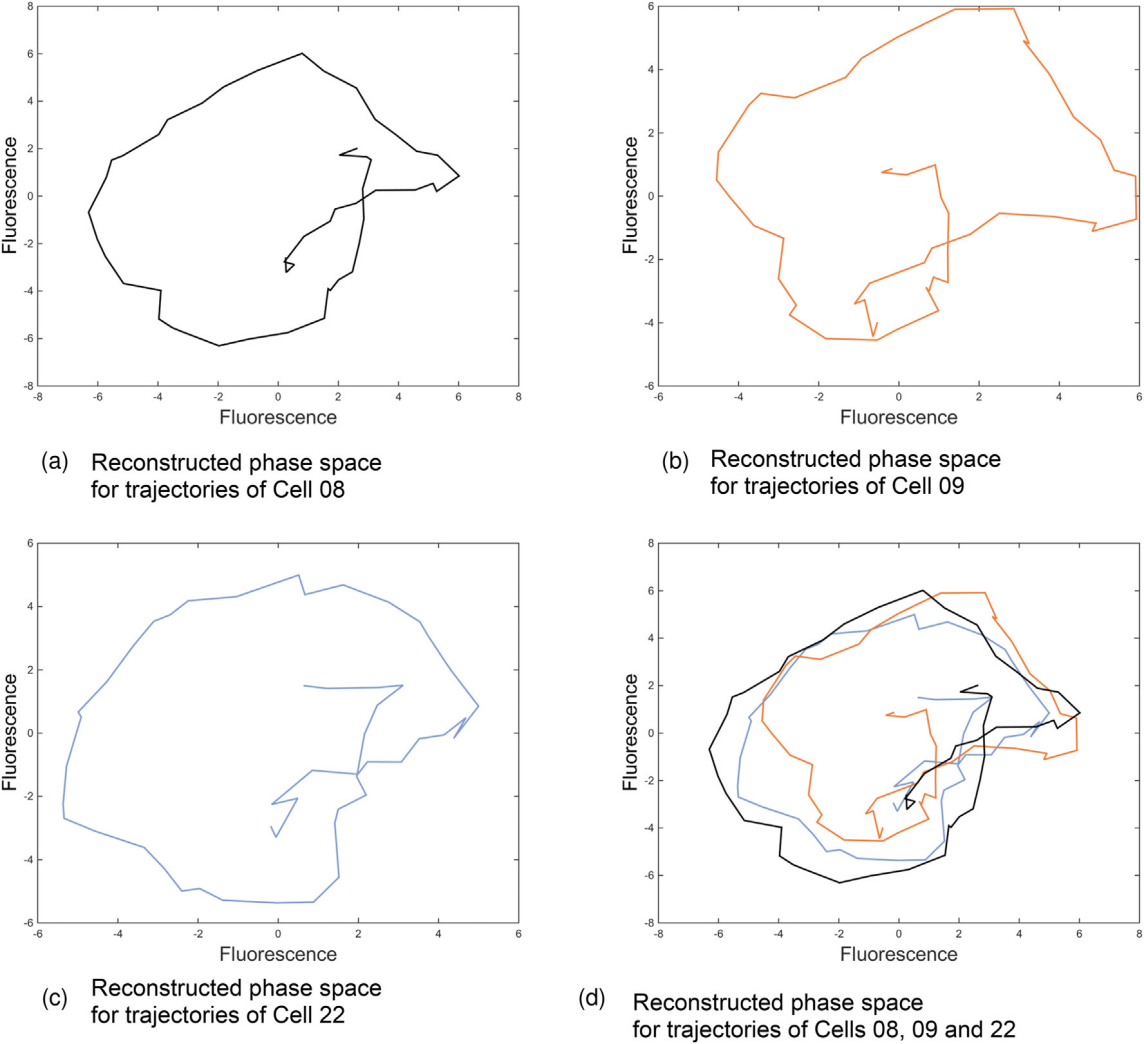
Reconstructed phase space of de-trended fluorescence data. Reconstructed phase space (as described in Material and Methods) plots of smoothed, de-trended fluorescence signal (arbitrary units) trajectory from images of cells 08 (A), 09 (B), 22 (C) and all three superimposed (D).

Obtaining de-trended oscillation data allowed us to reconstruct phase space of the fluorescence signal using Takens’ time-delay embedding method (Takens 1981). The resulting phase planes for all three cells (Figure 13A-C) were indicative of a periodic limit cycle i.e. a closed trajectory. This is consistent with the observed fluorescence dynamics resulting from changes in Ub-eGFP quantity. Figure 13D shows the overlaid, reconstructed phase space for the three trajectories for each of the 3 cells. Outliers can be attributed to the data points on the edges of the data set, such as the first and last data points, which cannot be processed in the same way as the intervening data points. The phase space for the 3 cells showed very similar overall pattern, suggesting the underlying causes of the oscillation were the same in each cell.

## 7 Conclusions

We have designed and simulated how an SGN encoding a GFP-based Gibson oscillator could function in *T. b. brucei* cells and then, informed by those simulations, successfully built and tested genes and transgenic cells to observe oscillations in their fluorescence with a period of 50 min, varying amplitude and linear growth trend. The data we have captured are consistent with an oscillator function but do not absolutely rule out alternative possibilities such as stochastic derepression of the tetO or limitations of the FILT experimental setup. If we assume the oscillations observed here are due to the intended function of the SGN, we can cautiously compare them with observations in other organisms harbouring engineered, oscillatory SGNs. The 50-minute *T. b. brucei* oscillatory period observed here is comparable to the oscillatory periods in the range of 10–40 min reported by Stricker et al. (2008), when implementing a similar SGN in *E. coli*, which also featured oscillating expression of one repressor and one GFP reporter ORF, but separately controlled by two identical copies of the repressible promoter. Notably, under certain conditions *E. coli* (Michelsen et al., 2003) and *T. b. brucei* (Woodward and Gull, 1990) share a similar duration, 45–65 min, for the C phase of their cell cycle, during which cells harbour two genome copies. Further investigation may reveal if these similarities in oscillation period and cell cycle phase duration are coincidental or significant.

Tigges et al. (2009) implemented an SGN for oscillatory GFP expression in a higher eukaryote, Chinese hamster ovary (CHO) cells, and shared data indicating approximately 10% of GFP-expressing cells exhibited oscillatory expression of the reporter, comparable with the 9.1% reported in this study. Given that CHO cells and procyclic *T. b. brucei* cells have comparable cell cycle durations, 10–12 h (Puck 1964) and 8.5 h (Woodward and Gull, 1990) respectively, it is notable that the SGN-directed oscillatory periods in these organisms differed significantly: 26 h and 50 min respectively. However, several features of the CHO SGN also differ significantly from the *T. b. brucei* SGN; the use of transcription activation and antisense RNA-based repression, distribution of genetic elements across three plasmids and implemention via transient transfection, so direct comparison of these oscillatory periods is difficult.

Future application of designed, genetic oscillation in *T. b. brucei* could be as a tool to investigate the dynamics and cellular impacts of antigen switching; swapping out the GFP reporter for a VSG variant. Given its ability to switch between different densely packed surface layers of antigen, *T. b. brucei* may in the distant future be modified for use as a vaccine format, either as dead cells or as a non-pathogenic, living commensal vaccine technology. Predictably designed and robust SGNs would be essential for any such future innovations.

This work provides a first iteration of the design-build-test-learn cycle as a platform to inform future cycles to further refine this synthertic gene network implementation in *T. b. brucei*. Future steps can include revised experimental setups that allow live video recording of fluorescence in living *T. b. brucei* cells and modifications to gene network design for improved robustness, to increase the percentage of transformant cells that exhibit the intended phenotype. Establishing *T. b. brucei* as a synthetic biology chassis in this way will help open up protozoan organisms more widely to synthetic biology approaches for investigating disease and building biotechnological tools.

## Declarations

### Author contribution statement

Yanika Borg, Sam Alsford & Darren N. Nesbeth: Conceived and designed the experiments; Analyzed and interpreted the data; Contributed reagents, materials, analysis tools or data; Wrote the paper.

Vasos Pavlika: Contributed reagents, materials, analysis tools or data; Wrote the paper.

Alexey Zaikin: Conceived and designed the experiments; Analyzed and interpreted the data; Contributed reagents, materials, analysis tools or data.

### Funding statement

Alexei Zaikin was supported by the 10.13039/501100012190Ministry of Science and Higher Education of the Russian Federation (N 075-15-2020-926).

Darren Nesbeth was supported by the 10.13039/501100000266Engineering and Physical Sciences Research Council (EP/I033270/1).

### Data availability statement

Data associated with this study has been deposited at https://doi.org/10.6084/m9.figshare.14845254.v3 and https://doi.org/10.6084/m9.figshare.14845035.v1.

### Declaration of interests statement

The authors declare no conflict of interest.

### Additional information

No additional information is available for this paper.
